# Ethical principles across countries: does ‘ethical’ mean the same everywhere?

**DOI:** 10.3389/fpubh.2025.1579778

**Published:** 2025-06-11

**Authors:** Agata Kaczmarek, Agnieszka Żok, Ewa Baum

**Affiliations:** ^1^Department of Social Sciences and the Humanities, Poznan University of Medical Sciences, Poznan, Poland; ^2^Doctoral School, Poznan University of Medical Sciences, Poznan, Poland; ^3^Division of Philosophy of Medicine and Bioethics, Department of Social Sciences and the Humanities, Poznan University of Medical Sciences, Poznan, Poland

**Keywords:** codes of medical ethics, ethical dilemmas, cross-cultural medicine, Poland, Ukraine, India, Thailand

## Abstract

Ethical principles serve as the foundation of healthcare practice, guiding medical professionals in their interactions with patients and shaping healthcare policies worldwide. However, the interpretation and application of these principles can vary significantly across different cultural and socio-political contexts. Understanding these variations is essential for enhancing cross-cultural healthcare practices. The aim of this review was to identify and show the differences and similarities in understanding and implementation of four ethical principles in Poland, Ukraine, India and Thailand. The PubMed database was searched for articles, which resulted in 16 papers about the principle of non-maleficence, 36 papers about the principle of justice, 79 on autonomy and 16 on beneficence, all of which were included in the review. The results revealed ethical dilemmas encountered in routine healthcare practice, highlighting both commonalities and distinctions across the analyzed countries. This analysis offers valuable insights into how ethical challenges are addressed within diverse healthcare systems, contributing to a deeper understanding of the needs of both patients and medical personnel.

## Introduction

The processes of globalization lead to the integration of international ideas and the convergence of diverse cultures, even within healthcare systems. In medical institutions, we encounter not only patients but also medical professionals who may be migrants from distant countries. This presents numerous challenges, including ethical ones, as the understanding of ethics is also influenced by cultural factors. Despite the existence of international codes of medical ethics, individual countries maintain their own codes, which are binding for practitioners within their jurisdictions. The articles within these codes are based on the four primary ethical principles: non-maleficence, beneficence, autonomy, and justice. However, the interpretation of these principles may vary across different cultural contexts. In our work we would like to answer the question: how are the four ethical principles (beneficence, non-maleficence, autonomy, and justice) understood in the medical environment in Poland, Ukraine, India, and Thailand?

We selected these countries for scientific analysis due to the significant influence of dominant religious traditions on their respective cultures. In Poland and Ukraine, Christianity plays a crucial role—Catholicism being the predominant denomination in Poland, while Orthodoxy is widely practiced in Ukraine. Similarly, in India and Thailand, culture is shaped by Hinduism and Buddhism, respectively. Buddhism, which originated from Hinduism, remains a fundamental aspect of spiritual and social life in Thailand, just as Hinduism does in India. The selection of these countries enables a comprehensive examination of the impact of diverse religious traditions on socio-cultural development and their interconnections.

To better understand the potential differences, it is essential to reflect on the question: What is culture?

Geert Hofstede’s concerns ways of thinking, feeling, and, consequently, the types of actions undertaken by individuals (1). According to Jerzy Kmita, culture is a set of normative and directive beliefs widely respected within a given community. This means that culture is a conceptual construct comprising a collection of fundamental beliefs that form a type of social consciousness. Beliefs about various matters shape the norms prevailing within a society and directly influence acceptable and prohibited actions. The ideals shared by a particular group form a value system, which serves as the cultural core of that community (2).

Building upon these considerations, we can briefly trace the historical development of medical ethics codes worldwide. One of the earliest known legal codes is the Code of Hammurabi, created in the ancient Near East during the 18th–17th centuries BCE. This code was based on a system of punishments and rewards for specific actions, including monetary compensation, and adhered to the principle that the punishment should correspond directly to the harm caused by the offense. Of the 282 articles in the Code, nine addressed procedures performed by physicians. The Hippocratic Oath, which emerged approximately 2,500 years ago, serves as the model for most modern codes of medical ethics in the Western world. It presents a set of principles that prioritize the welfare of the patient above all. Physicians are expected to take responsibility for their patients, society, and their own actions (3). The ethical foundations of medical practice were primarily based on the principles of the Hippocratic Oath until the formulation of the Georgetown Mantra. These principles emphasized beneficence and non-maleficence. Autonomy and justice were only introduced in 1979, completing the framework of the four principles of bioethics (4).

The foundational principles of medical practice in ancient India can be traced to Hinduism and its derivatives, Jainism and Buddhism. Buddhism, in particular, emphasizes the attainment of nirvana through the elimination of suffering. The earliest sacred texts of Hinduism, written in Sanskrit, are also regarded as the first sources of documented medical practice. These texts were introduced to India around 1,500 BCE during the Aryan invasion from Central Asia, when the Aryans settled in the northern regions of the country. A subsequent addition to the Vedic corpus, the Atharvaveda, serves as the primary source of medical knowledge from the Vedic period, which spanned the second to the first millennium BCE and lasted until the 6th century BCE. The methods of medical practice in later years are detailed in foundational Ayurvedic works such as the Caraka Samhita, Susruta Samhita, and Bhela Samhita, which are dated to approximately 600 BCE. These texts reflect an ethical approach that placed significant emphasis on the concept of the cycle of life, death, and rebirth. Early Vedic healers were drawn from priestly communities, and their medical practices were deeply rooted in the philosophical framework of the time. Later texts also exhibit numerous references to Buddhist philosophy (5). For example, in the Bodhicharyavatara by Śāntideva (600 CE), one finds the following passage: “Although I cannot directly experience another’s pain as my own, it is still a suffering they cannot endure… I must alleviate the pain of others as though it were my own, for it is suffering; I must extend kindness to others because they are living beings, just as I am.” This intertwining of medical practice with spiritual and philosophical traditions underscores the deeply ethical and compassionate foundations of ancient Indian medicine (6).

## Materials and methods

A literature review was carried out to analyze studies on the understanding and implementation of four ethical principles into medical practice in Poland, Ukraine, India and Thailand. The articles were acquired from PubMed database, using following Boolean combinations: ((autonomy) AND (poland)) AND (ethics), ((autonomy) AND (ukraine)) AND (ethics), ((autonomy) AND (india)) AND (ethics), ((autonomy) AND (thailand)) AND (ethics), ((justice) AND (poland)) AND (ethics), ((justice) AND (ukraine)) AND (ethics), ((justice) AND (india)) AND (ethics), ((justice) AND (thailand)) AND (ethics), ((nonmaleficence) AND (poland)) AND (ethics), ((nonmaleficence) AND (ukraine)) AND (ethics), ((nonmaleficence) AND (india)) AND (ethics), ((nonmaleficence) AND (thailand)) AND (ethics), ((beneficence) AND (poland)) AND (ethics), ((beneficence) AND (ukraine)) AND (ethics), ((beneficence) AND (india)) AND (ethics), ((beneficence) AND (thailand)) AND (ethics). The databases were searched between August and November 2024. The literature review applied a publication date limitation, including only studies published between 2014 and 2024.

**Figure 1:**
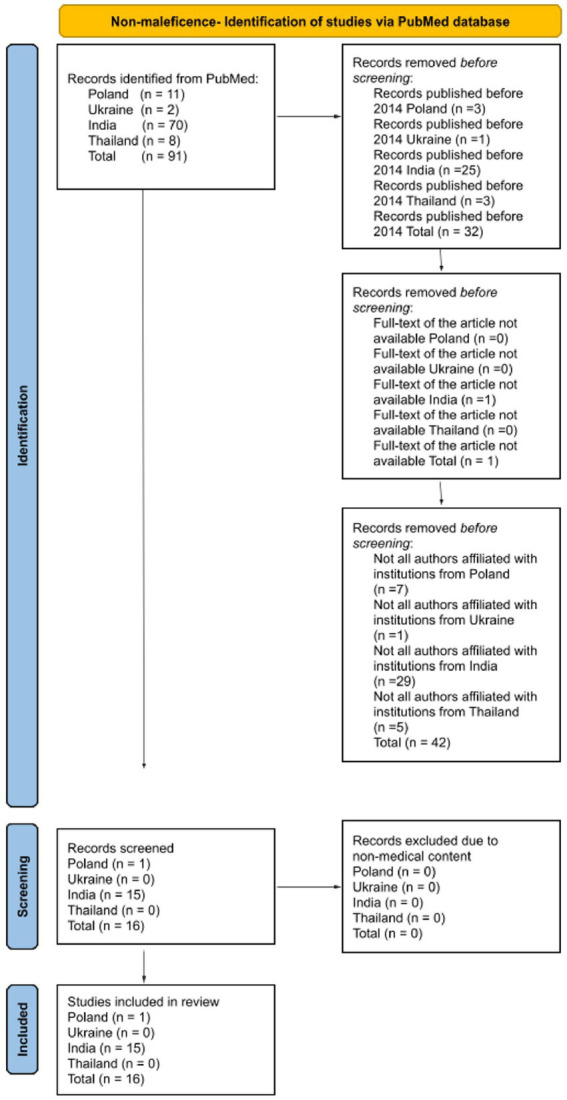
Diagram based on PRISMA 2020 showing the selection of articles for the literature review about the principle of non-maleficence.

**Figure 2:**
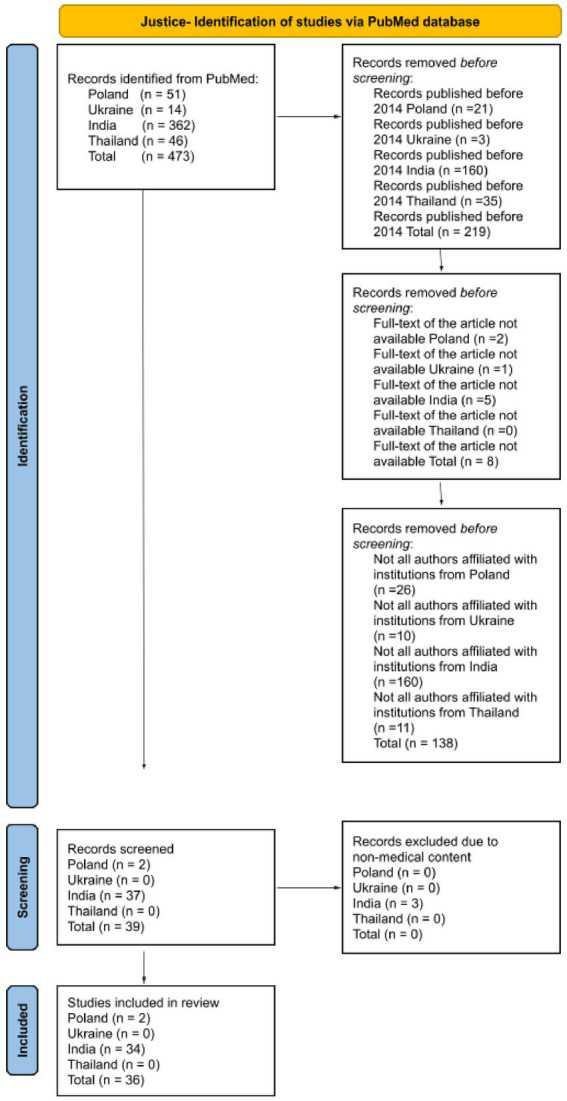
Diagram based on PRISMA 2020 showing the selection of articles for the literature review about the principle of justice.

**Figure 3:**
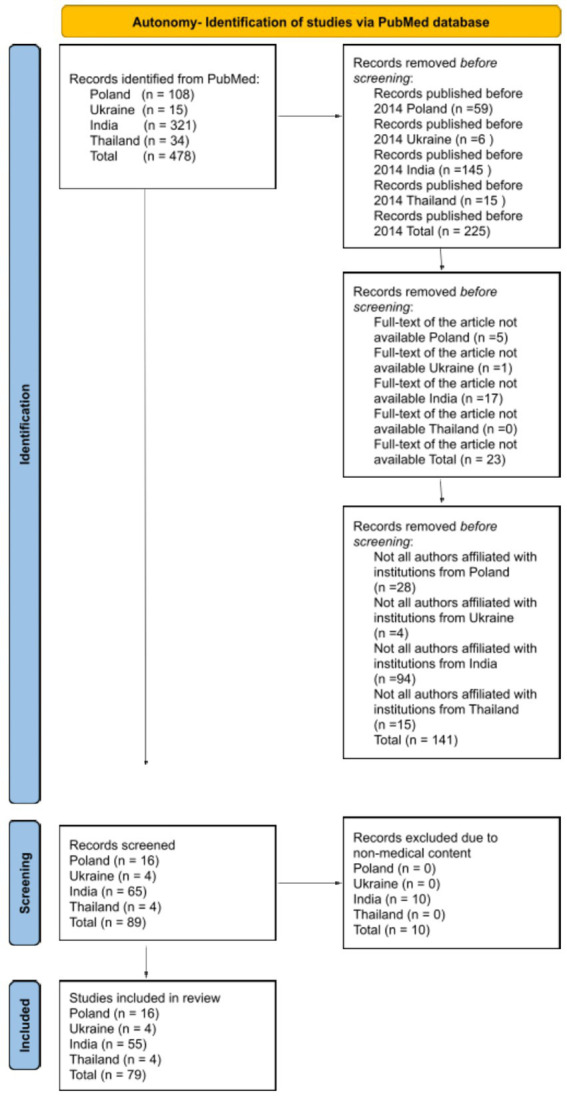
Diagram based on PRISMA 2020 showing the selection of articles for the literature review about the principle of autonomy.

**Figure 4:**
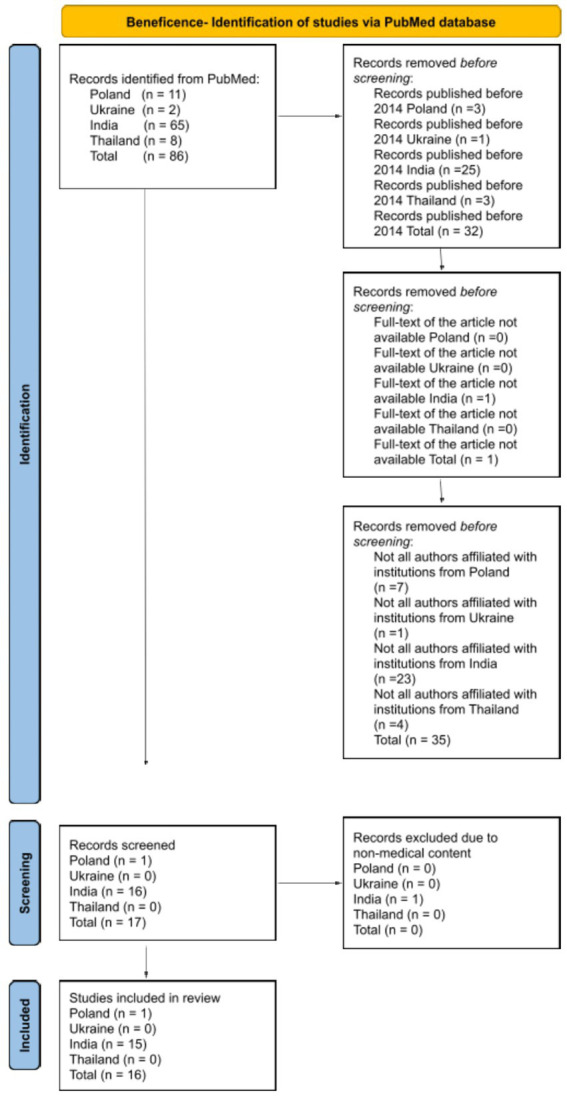
Diagram based on PRISMA 2020 showing the selection of articles for the literature review about the principle of beneficence.

The search was restricted to English-language articles focusing on the four ethical principles, as examined in scientific publications authored by researchers affiliated with institutions in one of the analyzed countries. All types of publications were included in the analysis, as not only original research articles reflect the understanding of ethical principles within a given cultural context, provided that the full text was available. The authors disqualified publications that did not cover the subject addressing ethical principles in relation to both patients and medical personnel. A detailed literature review methodology was meticulously outlined for each ethical principle, employing a diagram based on PRISMA 2020 to enhance the transparency of the analyses (Figures 1–4).

The primary search of databases resulted in 91 identified article records about the principle of non-maleficence. The initial review of studies, applying the exclusion criteria, resulted in the rejection of 32 articles due to their publication prior to 2014, one article due to the unavailability of its full text, and 42 articles because not all authors were affiliated with research institutions in one of the analyzed countries. A total of 16 studies were included for screening, and none were excluded due to non-medical content. All 16 studies were subsequently included in the final literature review.

The initial database search yielded 473 article records related to the principle of justice. After applying the exclusion criteria, 219 articles were discarded for being published before 2014, 8 articles were excluded due to the lack of access to their full text, and 138 articles were eliminated because not all authors were affiliated with research institutions in one of the studied countries. A total of 39 studies were selected for further screening, of which 3 were excluded due to non-medical content. Ultimately, 36 studies were included in the final literature review.

The initial search of the databases identified 478 articles pertaining to the principle of autonomy. Following the application of the exclusion criteria, 225 articles were removed due to their publication prior to 2014, 23 articles were excluded because their full text was not accessible, and 141 articles were eliminated as not all authors were affiliated with research institutions in one of the examined countries. A total of 89 studies were selected for further screening, of which 10 were excluded due to non-medical content. Ultimately, 79 studies were included in the final literature review.

The initial database search retrieved 86 articles related to the principle of beneficence. After applying the exclusion criteria, 32 articles were excluded due to their publication date being prior to 2014, 1 article was removed due to the unavailability of its full text, and 35 articles were discarded as not all authors were affiliated with research institutions in one of the countries under study. Seventeen studies were selected for further screening, with 1 being excluded due to non-medical content. In the end, 16 studies were included in the final literature review.

The majority of the analyzed articles published between 2014 and 2024 by Polish researchers appeared in the international journal Medicine, Health Care and Philosophy. In the same period, the highest number of analyzed articles authored by Ukrainian researchers were published in the Polish journal Wiadomości Lekarskie. Medical Advances. Similarly, the largest share of publications by Indian researchers during 2014–2024 were featured in the local journal Indian Journal of Medical Ethics. In contrast, articles by Thai researchers were published across various international journals, such as The Lancet HIV and BMC Medical Education, with none appearing multiple times in the analyzed dataset.

## Results

### Non-maleficence

Applying the previously described search criteria, one scientific publication from Poland and fifteen from India were selected, each addressing the understanding of non-maleficence as one of the four ethical principles. The publications concerning Ukraine and Thailand did not meet the established criteria.

In Poland, non-maleficence is primarily discussed in the context of medical treatment and scientific research. This principle requires physicians and researchers to avoid causing harm. It mandates assessing the risks and benefits of medical or research interventions to prevent inappropriate actions. This includes minimizing physical, emotional, and socioeconomic harm to patients or research participants. Nonmaleficence prohibits actions that cause intentional harm and ensures risks are proportionate to benefits, emphasizing respect for individuals’ well-being. It is closely tied to beneficence, forming a balance between preventing harm and promoting good while safeguarding participants’ dignity and autonomy (7).

From the Indian context nonmaleficence is also named as the principle of “first, do no harm.” It requires avoiding intentional harm or injury to patients and research participants (8, 9). Non-maleficence includes addressing medical errors, ensuring transparent and ethical handling of diagnoses, preventing harm during screenings, avoiding overdiagnosis, and mitigating unnecessary investigations or expenditures. What is more, doctors also should take care about effective communication and balanced decision-making (10). When treating a patient and considering additional therapeutic options, it is essential to account for long-term consequences. In some cases, refraining from certain procedures is necessary to ensure the patient’s well-being, particularly when the burden (non-maleficence) significantly outweighs the potential benefit (beneficence) (11). Doctors should not sell or promote agents or devices as being therapeutic without adequate evidence about the medical benefit (12). Non-maleficence is also evident in the practice of trainee therapists in clinical psychology. The use of certain therapeutic techniques raises concerns about the potential negative impact on, or decisions affecting, the client’s well-being (13). Rural areas in India present a distinct challenge both in terms of medical provision and the ethical approach to compromises made in the delivery of healthcare services. The National AIDS Control Organization may be invoking the principle of non-maleficence as justification for banning Unbanked Directed Blood Transfusion (UDBT), a decision influenced by the findings of a report on blood banks in India. This report highlighted the substandard quality of blood banks but did not address the practice of UDBT. Some authors argue that this decision is based on the absence of solid evidence, and that banning UDBT in emergency situations contradicts the principles of beneficence, justice, and autonomy (14). The principle of non-maleficence can conflict with beneficence, the obligation to act for others’ benefit, due to the inherent risks and benefits of medical interventions. Typically, beneficence takes precedence when a socially valuable outcome is at stake. However, certain authors argue that during the SARS-CoV-2 pandemic in India, inadequate post-marketing surveillance of vaccines and delays in publishing national data may have skewed public risk perceptions. This imbalance likely disrupted the ethical equilibrium between beneficence and non-maleficence (15). Healthcare decisions should prioritize the protection of patient safety, ensuring that no harm is inflicted upon individuals or society. For instance, emergency use authorizations for vaccines aim to safeguard vulnerable populations, such as pregnant women and children, from potential risks. Providing unrestricted access to vaccines and offering compensation for adverse events underscore a commitment to minimizing harm, addressing both health and financial risks. These measures reflect a careful balance between the broader societal benefits and the protection of individual safety, thereby upholding the principle of non-maleficence (16). It may be violated in a situation of vaccine safety, especially with regard to the oral polio vaccine (OPV). While OPV was cost-effective, it introduced risks such as vaccine-associated paralytic polio (VAPP) and prolonged shedding of neurovirulent vaccine viruses in immunocompromised individuals. In contrast, the inactivated polio vaccine (IPV) offered a safer and equally effective alternative. The use of OPV in India, without a thorough cost–benefit analysis or appropriate compensation mechanisms, raises significant ethical concerns. Public health decisions should not prioritize cost over safety, ultimately compromising the protection of public well-being (17). In the context of the animal bite case, non-maleficence requires that clinicians take necessary precautions to protect patients from harm, such as recommending a full course of post-exposure prophylaxis (PEP) even in the absence of documented history. However, this principle must be balanced with other ethical considerations, such as justice, since the unnecessary use of resources and the potential strain on the healthcare system may lead to harm in other areas, thus violating the principle of distributive justice (18).

When addressing the clinical trials, practitioners should thoroughly discuss the potential side effects and complications associated with the trial procedures prior to enrolling an individual in the study. At this stage, practitioners may also recommend alternative procedures or treatments that could be more advantageous for the patient (19). Researchers must consider whether intervening in a participant’s private life might cause more harm, especially in patriarchal, rural settings where societal norms could exacerbate risks. Safeguarding privacy and confidentiality is crucial, ensuring that any actions do not expose participants or their families to greater danger. This highlights the complexity of aligning non-maleficence with social justice in sensitive cultural environments (20).

The development of medical technologies and the storage of patient medical data pose new ethical challenges. The principle of beneficence supports the use of Electronic Medical Record System data in clinical and biomedical research for the benefit of individual patients and society. However, this principle can come into conflict with non-maleficence, particularly if sensitive health information or patient identities are exposed, potentially compromising patient dignity. To uphold ethical standards, it is imperative to ensure that patient identity and data remain secure and confidential throughout and following the research process (21).

The topic of abortion is a globally contentious issue that evokes strong emotions and represents a significant ethical dilemma. Indian physicians argue that the Supreme Court of India’s decision to deny a mother’s request to abort a 26-week-old fetus diagnosed with Down syndrome contradicts the ethical principle of non-maleficence. The ruling, grounded in the Medical Termination of Pregnancy Act of 1971, overlooked the mother’s autonomy, potentially exposing both the child and the parents to significant harm. The child may face a diminished quality of life due to inadequate resources, while the parents could experience moral distress and deteriorating mental health (22).

### Justice

Based on the specified search criteria, two scientific publications from Poland and thirtyfour from India were identified, all exploring the concept of justice as one of the four fundamental ethical principles. However, the publications related to Ukraine and Thailand did not satisfy the defined requirements.

According to Polish scientific publications, the principle of justice emphasizes the ethical obligation to ensure equitable access to healthcare benefits and fair distribution of burdens. It requires providing all patients with equal opportunities to access modern medical technology regardless of socioeconomic or geographic disparities. Justice also entails supporting diverse communication needs, whether verbal or through sign language. Patients should be fully informed about available solutions and communication options (7). The principle of justice emphasizes fairness in the distribution of research burdens and benefits among participants. Concerns arise when payment for participation disproportionately attracts individuals of lower socioeconomic status, potentially leading to exploitation and unequal benefit distribution. Justice requires avoiding exploitation by ensuring participants are not unduly disadvantaged due to poverty or urgency, and by addressing systemic inequities without exacerbating them through research practices. Ethical recruitment should not target vulnerable populations for convenience but reflect the scientific purpose of the study. Recompense for direct research-related costs serves to remove economic barriers. Justice thus ensures that all individuals have fair access to the benefits of research without being unfairly burdened (23).

In the review of Indian literature, a significant number of publications were excluded as the only connection to the term “justice” was the approval of the bioethics committee named “Committee for the Purpose of Control and Supervision of Experiments on Animals (CPCSEA), Ministry of Social Justice and Empowerment, Government of India.” In the included articles the principle of justice emphasizes equitable and fair treatment for individuals (8, 24).

All patients deserve equal respect and fair treatment, irrespective of their socioeconomic status, caste, gender, religion, or nationality (25). Justice mandates removing systemic barriers that marginalize specific groups, such as transgenders and economically disadvantaged populations (26). Healthcare providers should distribute services proportionate to individuals’ needs (27). For persons with disabilities reducing barriers and ensuring liberal and preferential access to healthcare is needed (28). A social justice-based approach emphasizes enhancing the quality of end-of-life care for individuals living with dementia while upholding their choices, autonomy, and dignity (29). In clinical research, fair treatment includes sharing both risks and benefits equitably among all trial participants (19). Educating them about the procedure and managing their expectations are also essential components. The concept of clinical equipoise ensures that no group is intentionally subjected to inferior treatment, safeguarding fairness and preventing exploitation (30). Health systems research in low- and middle-income countries needs the integration of local values and social justice into public health policies (31). Circumstances and moral relativism can complicate the application of justice. Community health workers may face challenges in upholding justice due to their personal vulnerabilities, societal pressures, or the lack of institutional support (32). The ethical discussion surrounding unbanked directed blood transfusions (UDBTs) in rural healthcare demonstrates justice as the equitable allocation of life-saving resources in underserved areas. The principle of justice supports the adoption of appropriate technologies like UDBTs to bridge systemic gaps in emergency healthcare, ensuring that populations have fair access to critical medical interventions. This highlights the need for context-specific solutions (14). Justice calls for systemic reforms to combat inequities in access to care, affordability, and the availability of resources, particularly in low- and middle-income countries. It also includes public policy measures like taxing unhealthy products and promoting healthier lifestyles while fostering innovation and collaboration for low-cost solutions (33). Favoritism or differential treatment of patients based on their socioeconomic status violates the equitable distribution of scarce medical resources. All patients, irrespective of their affluence or bargaining power, should receive unbiased and evidence-based care (34). The idea of Ayushman Bharat programme adresses the challenges of achieving equity in a healthcare system. While this programme aims to provide financial protection to economically disadvantaged families for secondary and tertiary care, it risks violating the principle of justice if it does not prioritize universal basic health insurance (35). According to some authors, doctors are encouraged to reduce fees or offer free care to impoverished patients, highlighting fairness in healthcare access (36). Another idea for reducing barriers to accessing the legal and healthcare systems is the use of videoconferencing tools (37).

The COVID-19 pandemic was not only a challenge for the healthcare system but also for ethics and social inequalities. In India, while the authorities and police employ stringent measures with the vulnerable sections, people of certain affiliations have been able to conduct marriages and other ceremonies. This violates the principle of social justice (38). Equitable vaccination distribution during emergencies, such as the COVID-19 pandemic, exemplifies the application of justice in action (16). Healthcare workers and vulnerable populations were prioritized without neglecting broader societal needs (39). Also wealthier nations should not be disproportionately benefiting by vaccinations programs (15, 40).

Public health professionals must prioritize equally significant health issues, such as immunization and tuberculosis control, leading to potential inequities (41). Global distribution of vaccines is challenging. The principle of justice was not upheld by the promotion of the oral polio vaccine (OPV) in low-income countries, despite its lower efficacy compared to the inactivated polio vaccine (IPV) used in wealthier nations (17). In the clinical management of rabies re-exposure cases, the wastage of anti-rabies vaccines and the associated opportunity costs borne by health systems and patients may occur due to excessive vaccination. Justice calls for optimizing resource allocation to balance individual patient care with broader societal needs, ensuring that resources are used efficiently (18).

New technologies are not only an organizational challenge, but also an ethical one. Patients should be educated about the advantages of electronic medical records systems and assured of confidentiality and privacy. Justice involves respecting patients’ rights to informed decision-making based on equal access to knowledge (21).

In the field of laboratory medicine, ensuring equitable access to diagnostic resources and preventing resource wastage through unnecessary investigations are essential. It also involves maintaining fairness by resisting external pressures to favor influential individuals at the expense of other patients (10). The National Medical Commission Act, 2019 illustrates a gap in procedural justice by unequally empowering doctors and patients in grievance redressal mechanisms. While medical professionals can appeal decisions through appellate fora, aggrieved patients lack similar avenues (42).

The principle of justice is also denied in the context of adolescent autonomy under India’s Protection of Children from Sexual Offences Act (POCSO). Criminalizing consensual adolescent sexual activities ignores their evolving capacities and autonomy, subjecting them to legal consequences that may unjustly impact their futures. Justice, in this instance, calls for a nuanced approach that balances protection with respect for adolescents’ rights (43). The alliance of neoliberal economic policies with conservative and religious ideologies has undermined gender and health justice by reversing established rights. Justice demands addressing these systemic drivers and protecting the autonomy and rights of vulnerable populations, particularly women (44). The right to abortion as a core element of reproductive freedom. Some couples delay access to medical termination due to socioeconomic status or geographic location. It underscores the systemic injustice faced by those from less privileged backgrounds compared to affluent individuals (22). India needs not only the formulation of progressive laws but also their practical enforcement, addressing the root causes of gender-based violence and ensuring accountability across multiple sectors (45). There is still a need for the recognition and redress of caste-based discrimination in professional and educational institutions. The evolving, covert forms of discrimination—such as bureaucratic delays and exclusion from training—necessitate systemic reforms to identify and address these injustices (46).

### Autonomy

Using the defined search parameters, a total of sixteen scientific publications from Poland, four from Ukraine, fifty-five from India and four from Thailand were identified. All of these works examine autonomy as one of the core ethical principles.

From a perspective shown in the Polish literature, the principle of autonomy demands structured mechanisms such as informed consent, clear communication, and tolerance of diverse viewpoints (47). Theoretically, autonomy is inherently linked to broader societal norms and values, such as tolerance and respect for diversity (48). Patients often lack sufficient understanding of informed consent documents, which compromises their ability to make autonomous decisions (49). For younger adults, autonomy is closely linked to independence and personal recognition, while for older adults, it aligns with independent thinking and rejection of humility. These findings suggest that the principle of autonomy is not monolithic; it is shaped by individual values and sociocultural contexts, requiring tailored approaches in its implementation (50). Actively supporting autonomy contributes to psychological and social well-being (51). Autonomy, understood as the ability to make choices and decisions, is a cornerstone of positive aging (52). While adults may exercise full autonomy, children’s autonomy is inherently limited. Decisions regarding interventions such as cochlear implants often require balancing the child’s developmental needs with their right to participate in decision-making. Postponing decisions to honor the child’s autonomy may conflict with the urgency of early medical action necessary for optimal outcomes. This dilemma illustrates the tensions between respecting autonomy and ensuring beneficence (7). Nurses demonstrated greater acceptance of adult patients’ autonomous decisions than those involving children, revealing a paternalistic inclination in life-threatening situations (53).

In the case of childhood vaccinations we can see a conflict between individual autonomy and public health mandates. Informed consent, which is integral to respecting autonomy, becomes problematic under mandatory vaccination policies. When parents are required to sign a consent form under the threat of legal consequences, the process lacks genuine voluntariness, rendering the consent ethically questionable (54). Penalties for refusing vaccinations (as practiced in Poland), may violate the principle of autonomy. However, imposing financial responsibility for treating preventable diseases in unvaccinated individuals might balance public health needs with respect for individual freedoms (55).

Payment for research participation is an ethical dilemma. While attractive payments risk undermining autonomy by influencing individuals to act against their better judgment, providing adequate compensation can enhance autonomous decision-making (23). Respect for the patient’s autonomy entails obtaining informed consent for medical or therapeutic interventions and his right to withdraw at any stage. This underscores a partnership model in healthcare, where professionals must ensure clear communication, safeguard patient privacy, and respect their decisions, even if those decisions differ from professional opinions (56). The persistence of paternalistic models in Polish healthcare, where informed consent is often treated as a formality rather than an ethical imperative, highlights systemic challenges (57). The ethical doctrine of autonomy emphasizes the patient’s right to know and, conversely, the “right not to know,” both of which are seen as extensions of their ability to make autonomous choices. The right not to know is argued to protect individuals from psychological distress or societal consequences that may arise for example from unwanted genetic knowledge (58). Dilemmas arise when patient autonomy conflicts with medical obligations, as illustrated in the case of Jehovah’s Witnesses refusing blood transfusions. Respecting autonomy in such scenarios demands navigating complex ethical tensions between honoring patients’ religious convictions and fulfilling the duty to preserve life (59).

Autonomy applies only to individuals deemed competent—those capable of rational thought and self-awareness. This criterion is crucial, as incompetency requires surrogate decision-making mechanisms, such as judicial consent for incapacitated adults in Poland. Professionals must exercise independent judgment within their domain of expertise, particularly in multidisciplinary teams. Infringements on professional autonomy can lead to diminished self-esteem, burnout, and reduced quality of care (60).

From the Ukrainian perspective the principle of autonomy underscores the importance of respecting individual rights in medical decision-making. It also highlights the unique socio-cultural and legal challenges that shape its implementation.

The conflict between respecting individual autonomy in making end-of-life decisions and societal or legal constraints on euthanasia is a profound issue in Ukraine. It is important to balance the right to end suffering with the risk of undermining the practice of palliative care and potentially increasing involuntary deaths (61).

While Ukraine adheres to the principle of reproductive autonomy as part of human rights, societal influences, particularly Christian moral values, contribute to the cautious approach to reproductive interventions. The importance of informed consent in reproductive healthcare, including posthumous reproduction and reproductive rights for individuals with disabilities, is emphasized (62). Interventions like circumcision cannot be viewed as autonomous choices even in refugee groups among which such practices are culturally accepted. They result in significant health consequences and societal harm. The medical and legal community must intervene in such practices to protect the individual’s bodily integrity (63). In the context of digital pathology in oncology patients are entitled to be informed about AI’s capabilities and limitations, as well as privacy protections (64).

Understanding the principle of autonomy in the Indian context reveals a complex interplay between cultural, ethical, and legal considerations (65). Autonomy is defined as the right to self-determination and informed decision-making (66).

While the principle of autonomy is enshrined in ethical guidelines and institutional frameworks, its application is mediated by cultural norms, societal hierarchies, and the operational realities of healthcare delivery (67). Especially in the rural areas there is a dynamic interplay between respecting individual rights and navigating the constraints imposed by traditional social structures (68).

In clinical psychology, trainee therapists often encounter ethical dilemmas when patient autonomy conflicts with the therapist’s personal or moral beliefs. This highlights the importance of respecting client autonomy in setting therapeutic goals and making life decisions, despite personal disagreements (13, 69).

Physician-assisted suicide in advanced dementia illustrates the profound challenges to autonomy in situations involving impaired cognitive capacities (29). While autonomy theoretically hinges on an individual’s ability to make voluntary, informed, and reasoned decisions, factors such as undue influence, depression, and compromised judgment complicate this principle in practice (70, 71). Practices like “Do Not Resuscitate” (DNR) orders and euthanasia are areas where autonomy conflicts with medical duty and societal norms. While autonomy allows patients to refuse life-sustaining treatment, the absence of clear legal recognition for DNR or advanced directives complicates the implementation of their wishes (72, 73). The legal recognition of living wills signifies progress toward acknowledging patients’ autonomy (74). However, inconsistencies in legislation and ethical dilemmas associated with their application reveal gaps in effectively translating this principle into practice (75).

In pediatric healthcare, the principle of autonomy is inherently limited, as children cannot make independent medical decisions (76). Guardians or parents act as proxies, tasked with promoting the child’s welfare while aligning with informed consent principles. This practice reinforces autonomy as a protective framework, ensuring decisions reflect the child’s best interests within the constraints of their developmental capacity (77). The legal framework governing adolescent autonomy, particularly concerning sexual and reproductive rights, demonstrates the limitations of current policies. The Protection of Children from Sexual Offences (POCSO) Act criminalizes all sexual activity under the age of 18, failing to account for consensual, non-exploitative relationships. This blanket criminalization disregards the evolving capacity of adolescents to make informed decisions about their bodies, effectively curtailing their autonomy (44, 78).

Public health interventions in India, such as those implemented during the COVID-19 pandemic, have raised critical questions about the limits of individual autonomy in the face of collective health concerns. The debate over forced alcohol abstinence during lockdown illustrates the tension between public health imperatives and individual rights. While certain measures, such as quarantine, may be justified as proportional restrictions, others, like prohibition policies, risk being perceived as paternalistic or rooted in moralistic values (79). The ethical implications of convalescent plasma therapy during the COVID-19 pandemic further highlight the intersection of autonomy with external influences. Political advocacy and commercialization can impede genuine autonomy by introducing biases or coercion into decision-making processes, both at individual and institutional levels (80). Mandatory vaccination policies, while pursued for public health, must satisfy the three-pronged test of legality, necessity, and proportionality to be considered constitutionally valid (81). This demonstrates that the state’s power to impose health-related mandates is circumscribed by the individual’s right to autonomy and privacy. The exclusive adoption of the Oral Polio Vaccine (OPV) in India, despite safety concerns such as vaccine-associated paralytic poliomyelitis (VAPP), exemplifies a policy that compromised individual autonomy (82). The lack of parental choice between OPV and the Inactivated Polio Vaccine (IPV) denied families the opportunity to make decisions about their children’s health (17). In situations like rabies re-exposure treatment, decisions that disregard patient history and preferences may further erode autonomy. While such actions may not overtly violate public health imperatives, they undermine the ethical principle of respecting the patient’s right to informed decision-making (18). Also mandatory food fortification can undermine autonomy by removing individual choice, reflecting a paternalistic approach that conflicts with personal freedoms (83).

In the context of antimicrobial stewardship, the principle of autonomy is particularly affected by social determinants and healthcare inequities. Interventions to regulate antimicrobial use, aimed at preventing resistance, sometimes restrict the autonomy of both prescribing physicians and patients. This is especially true when clinical diagnoses are unclear, and prescribing decisions are based on professional judgment rather than explicit clinical guidelines (41).

In the context of maternal healthcare, the principle of autonomy takes on a transformative role in empowering women. Research indicates a strong association between higher levels of women’s decision-making autonomy and increased utilization of antenatal and postnatal care services (84–86). Experience of low autonomy also correlates with high levels of depression (87). In the case of mid-life fertility treatments, physicians face ethical dilemmas in balancing respect for patient autonomy with their professional judgment about the efficacy and outcomes of treatments. Patients, often influenced by the stigma of infertility, may insist on pursuing interventions despite low chances of success (88). Women’s ability to make autonomous reproductive decisions is frequently overridden by the prioritization of familial, societal, or even legal considerations (89). Selective sex abortions, stigma surrounding abortion, and legal resistance to terminations beyond 20 weeks further demonstrate how autonomy is subordinated to cultural and institutional imperatives (90). Decisions often require spousal or familial consent, reflecting limited agency for women. For instance, requiring a husband’s approval for abortion services undermines a woman’s ability to make autonomous reproductive choices. Similarly, the prioritization of male authority, as observed in decisions regarding contraceptive implantation, reveals entrenched gender biases (91, 92). The Medical Termination of Pregnancy (MTP) Act grants significant decision-making power to medical practitioners rather than the women seeking abortions (93). The law’s focus on population control rather than individual choice (94).

In the context of clinical research, autonomy is upheld through the practice of informed consent, requiring that patients be provided with comprehensive information about medical procedures, associated risks, and available alternatives (19, 95, 96). Many participants in India, due to limited literacy or understanding of complex medical information, rely on the recommendations of healthcare providers rather than exercising independent decision-making (97). The reuse of biological samples without explicit consent poses ethical challenges, as it risks undermining trust in science and patient autonomy. A balance between respecting autonomy and fostering altruism and solidarity allow for future use of samples under ethical oversight (10).

Autonomy in India is often compromised by cultural norms and the preference for paternalistic approaches. The informed consent must come directly from the patient, not from third parties like family members (98). The familial influence on medical decision-making in India, as opposed to the individualistic focus of Western medical ethics, further complicates the notion of autonomy. The involvement of family members in the patient’s treatment decisions is common. The treatment team feels accountable not only to the patient but also to the patient’s family. This has profound implications for patient autonomy, especially in cases where patients are influenced by family members to seek or refuse treatment. This dynamic is particularly visible in cases involving substance use disorders, where family members often pressure the patient into treatment, even against the patient’s will (99).

Respect for autonomy is an ethical principle that obliges healthcare providers to enhance the patient’s capacity for decision-making by providing information about medically justified treatment alternatives for the patient’s condition (22, 100). In the case of cosmetic limb lengthening if a patient of average height requests height enhancement surgery, the physician cannot ethically refuse the request. Denying this request would constitute a violation of the patient’s right to autonomy (101). Practices that restrict patients’ choices, such as the sale of medications directly through clinics disrupt autonomy. Patients’ right to access cost-effective and alternative treatments is compromised when registered medical practitioners (RMPs) prioritize the sale of proprietary products (12, 102). The hope for a better future is that awareness of autonomy among medical students correlates with better clinical reasoning. It underscores the importance of good understanding of autonomy in professional development (103–105).

Advancements in neurotechnology, such as brain-computer interfaces, deep brain stimulation (DBS), and functional MRI (fMRI), have amplified concerns about individual autonomy. While these technologies hold potential for treating neurological conditions like Parkinson’s disease, depression, and obsessive-compulsive disorder, they also introduce ethical dilemmas. Autonomy is particularly contested in scenarios where cognitive functions are impaired. Moreover, fMRI and similar technologies challenge autonomy through potential breaches of privacy (39). Decoding brain activity for therapeutic or legal purposes could lead to exploitation, such as misrepresentation in court cases or manipulative external control over an individual’s actions (106). Also introduction of artificial intelligence (AI) can cause loss of autonomy in clinical settings for both healthcare providers and patients (107).

The recognition of autonomy as a key factor in promoting professional and personal well-being is an important aspect of understanding how autonomy is valued in Thai healthcare settings. In the group of residents females who report higher levels of autonomy compared to their male counterparts, also experience greater well-being. Additionally, factors such as sleep and regular exercise are associated with a greater sense of autonomy. This suggests that autonomy is not only a theoretical or legal concept but is also linked to personal lifestyle factors (108). Comparing the views of Thai older patients and nurses on end-of-life care reveals discrepancies between the nurses’ perceptions and patients’ actual wishes. While healthcare professionals may understand the theoretical importance of autonomy, they may sometimes misinterpret or overestimate the desire of patients to exercise this autonomy in end-of-life decisions. This disconnect between professional perceptions and patient realities calls for greater communication and respect for individuals in medical practice (109). In Thailand the preference for voluntary euthanasia reflects a cultural and ethical commitment to personal autonomy. Dignity in death is achieved when a person has the freedom to make a final decision regarding their life. This aligns with the global ethical principle that individuals should have control over their own bodies and life choices, especially in the context of terminal illness or unbearable suffering. Autonomy in the Thai context may be viewed more positively when individuals actively choose to end their lives rather than when decisions are made on their behalf (110). The World Health Organization’s “test-and-treat” strategy for HIV treatment can give potential harm and loss of autonomy due to overtesting and overtreatment. This situation illustrates the tension between public health goals and individual autonomy, where patients may feel pressured into treatment options without the full freedom to make informed decisions (111).

### Beneficence

Using the search criteria outlined earlier, three scientific publications from Poland, sixteen from India, and one from Thailand were identified. Each publication explores the concept of beneficence as one of the four fundamental ethical principles. However, the publications related to Ukraine did not satisfy the specified requirements.

In Poland, the principle of beneficence is understood as a fundamental ethical obligation requiring physicians to act in ways that promote the welfare of patients. They should be provided with alleviating conditions that may lead to harm. Unlike the principle of non-maleficence, beneficence imposes positive duties to actively benefit individuals and enhance their well-being (7). In the realm of biomedical research, beneficence is often framed within the broader context of “social beneficence,” which underscores the importance of contributing to societal good. Research practices are justified ethically by their potential to generate generalizable knowledge that leads to safer and more effective diagnostic, preventive, and therapeutic measures. This perspective also highlights the role of compensating research participants as an ethically sound practice, given its ability to enhance recruitment and retention, thereby advancing the collective benefits derived from research (23). Furthermore, the principle of therapeutic beneficence is central to research involving human subjects. It binds physician-researchers to a fiduciary duty to safeguard the health interests of participants, ensuring that risks are only justified when outweighed by potential therapeutic benefits. At the same time, participants are regarded as ends in themselves, emphasizing that they should never be treated merely as means to achieve scientific objectives (112).

In Thailand, one article meeting the criteria for a review paper describes The working experience of nurse anesthetists with beneficence for patients. Healthcare professionals should communicate and listen to patients with compassion. Nurses should be considerate and knowledgeable. They must prioritize standard procedures, effective team communication, and patient safety to ensure a productive and harm-free work environment (113).

In the research articles on the Indian perception of the beneficence it is integral to clinical practice, therapeutic interventions, and research endeavors. This ethical imperative requires practitioners to prioritize the welfare of patients while balancing potential benefits against risks (8, 19). In clinical settings, beneficence mandates that medical interventions prioritize patient welfare. The decision-making process requires clinicians to evaluate patient prognosis through systematic steps, such as assessing deteriorating health indicators, to ensure the decision aligns with the patient’s best interests (11). Antibiotic prescribing practices for the patient’s best interest can conflict with long-term public health goals such as reducing antimicrobial resistance. Ethical challenges arise when patient demands, socioeconomic constraints, and physician biases lead to overprescription, requiring a balance between patient benefits and public health concerns (34).

Sometimes, also during the management of pregnancy, a physician encounters unique ethical challenges. In the treatment of supraventricular tachycardia in a twin pregnancy, the well-being of the affected fetus with potential risks to the healthy twin and the mother should be balanced. This ethical dilemma emphasizes the physician’s duty to make evidence-based decisions that prioritize clinical benefits, even when such decisions may oppose the patient’s autonomy (22, 100, 114). Approximately 8.5% of clinical psychology trainee therapists reported ethical dilemmas related to this principle. Underscore the necessity for therapists to critically evaluate the consequences of their interventions to ensure that their actions contribute positively to clients’ mental health and well-being (13). Also in laboratory medicine, physicians are encouraged to go beyond diagnosis. They should provide comprehensive advice, recommend further consultations, and ensure critical information is conveyed effectively to prevent harm. Fee-splitting practices, which increase patient costs, are critiqued as a violation of beneficence because they undermine patient welfare by prioritizing financial incentives over ethical responsibilities (10). Furthermore, the principle of beneficence extends beyond individual patient care to broader public health contexts, such as managing substance use disorders, where healthcare professionals are tasked with addressing both immediate patient needs and long-term societal impacts, such as stigma and resource constraints. In such cases, beneficence requires careful consideration of the cultural and systemic factors that shape healthcare decisions (41). Similarly, in public health initiatives like responding to a gastrointestinal illness outbreak, beneficence calls for swift, evidence-based actions to mitigate harm and protect the well-being of affected individuals, underlining the responsibility of healthcare organizations to prioritize patient welfare in both clinical and community settings. Ultimately, beneficence underscores the ethical imperative to act in ways that promote patient welfare while balancing individual rights, societal needs, and available resources (9). The COVID-19 pandemic highlighted the ethical responsibility to make decisions that prioritize patient benefit. There was the tension between beneficence and non-maleficence in the context of COVID-19 care protocols. Placing every critically ill patient on ventilators may not align with the principle of benefit if the overall well-being and comfort of the patient will not be prioritized (82). The discussion of beneficence can be extended to public health interventions, such as vaccination campaigns. While beneficence supports the promotion of societal health benefits, it often conflicts with non-maleficence and informed consent when risks are inadequately communicated (15). Recognizing the novel and uncertain nature of the virus and its vaccines, the Government of India implemented extensive training at multiple administrative levels to equip healthcare professionals with the necessary skills and knowledge. This proactive approach underscores the commitment to beneficence by striving to maximize the benefits of vaccination while addressing potential risks associated with limited long-term efficacy data (16). The application of the principle of beneficence in scientific research can be linked to its generating good not only for the participants but also for society. This perspective emphasizes that the outcomes of research must extend beyond individual cases to contribute to broader social good. Researchers should publish findings, including negative ones, in reputable scientific journals to prevent resource wastage and ensure the availability of data for future inquiries (10, 115). In clinical trials beneficence mandates researchers and stakeholders prioritize participants’ well-being by promptly reporting and managing adverse events and serious adverse events. This responsibility is upheld through ethical and legal frameworks, ensuring that participants’ safety is safeguarded and any potential harm is minimized (116).

## Discussion

The exploration of ethical principles in healthcare across diverse cultural settings uncovers intriguing subtleties. Although foundational principles such as autonomy, beneficence, non-maleficence, and justice are universally recognized, their interpretation and practical implementation sometimes differ according to cultural norms and values. The most significant differences can be observed in the approaches to autonomy and justice.

In Poland, the principle of autonomy emphasizes structured mechanisms like informed consent, clear communication, and respecting diverse viewpoints (7, 47, 50). However, there are challenges with patients fully understanding consent documents, which can undermine their ability to make autonomous decisions. Autonomy is also shaped by individual values and social contexts, requiring tailored approaches. The majority of scientific articles authored by Polish researchers focused on the principle of autonomy, which aligns with the prevailing trend of patient-centered medicine in Poland. This focus reflects the growing emphasis on incorporating patients into the decision-making process regarding their healthcare. Recent developments in the healthcare system aim to enhance patient involvement in treatment choices, emphasizing respect for their autonomy and ensuring that they are active participants in their care (23, 54). Furthermore, patient organizations in Poland have gained significant influence, playing a pivotal role in shaping health policy and advocating for changes that prioritize patient rights and well-being. This shift represents a broader movement toward a more patient-centric approach in both clinical practice and healthcare policy formation in the country (117). The Ukrainian perspective highlights the tension between respecting individual autonomy in end-of-life decisions and legal constraints on practices like euthanasia. There’s a need to balance the right to end suffering with the risk of undermining palliative care (61). The majority of articles from Ukraine included in the literature review focused on the principle of autonomy. Most of the publications addressing other ethical principles were excluded due to their multi-center nature. This reflects Ukraine’s strong inclination towards international collaboration, yet, within the context of this study, it may limit the ability to distinctly delineate the cultural context. The country’s desire to align with international trends is also evident in the Ethical Code of Ukrainian Doctor, which was developed based on international documents. While this alignment demonstrates Ukraine’s commitment to global standards, it may also present challenges in capturing the unique cultural perspectives that shape ethical decision-making within the local healthcare system.

Autonomy in reproductive healthcare is also influenced by cultural and religious factors. In the Indian context, the principle of autonomy is often complicated by strong family involvement in medical decision-making. The treatment team feels accountable not just to the patient, but also to the patient’s family. This can undermine patient autonomy, especially in cases like substance abuse where families may pressure the patient into treatment against their will (99). Respecting autonomy is an ethical imperative, but it can conflict with other principles like beneficence and justice. For example, mandatory vaccination policies may violate autonomy, even if pursued for public health (17). Balancing individual rights with societal needs is an ongoing challenge. The application of justice highlights issues of equitable access to healthcare, fair distribution of research benefits, and addressing systemic barriers that marginalize vulnerable groups. In India, there are concerns around favoritism, differential treatment based on socioeconomic status, and the need for universal health coverage (12).

This study included only five research articles from Thailand. Unfortunately, the majority of the articles retrieved did not meet the criterion of being single-center studies conducted within the analyzed country. This limitation suggests that there may be a gap in the available literature in English specifically addressing ethical principles within the context of Thailand’s healthcare system.

According to LeDoux and Mona, understanding the differences between cultures is essential for building culturally competent healthcare systems (118, 119). Also Castaneda-Guarderas work showed that respecting diverse religious, social, and cultural needs, ultimately improving quality and effectiveness (120). In his work, Turner criticizes Tom Beauchamp and James Childress, advocates of the principled approach to morality, for assuming the existence of a stable, universal moral order. He argues that they view society as a monolithic construct, overlooking the significant influence of religion and culture in shaping ethical understanding (121). Acknowledging cultural diversity, our paper seeks to demonstrate how these ethical principles are interpreted differently across countries and how their meanings may vary depending on context.

The literature search conducted in this study revealed a limited number of original research articles on the understanding and application of ethical principles in clinical practice within the analyzed cultural contexts. This finding highlights a significant gap in the existing body of knowledge. Consequently, the authors argue that this area requires more in-depth exploration to better understand how ethical principles are perceived and applied across different cultural settings. Further research is essential to provide a comprehensive understanding of how these principles are integrated into clinical practice, as well as to identify potential cultural variations and their impact on ethical decision-making in healthcare.

## Conclusion

This study examines how core ethical principles: autonomy, beneficence, non-maleficence, and justice are interpreted within diverse cultural contexts. The findings reveal that while these principles are universally acknowledged, their implementation is heavily shaped by cultural, social, and institutional factors. Autonomy emerged as the most frequently discussed principle. In Poland and Ukraine it reflects a broader trend toward patient-centered care and alignment with international ethical standards. However, challenges persist, including limitations in patient comprehension, legal constraints, and the influence of family dynamics, particularly evident in India. The Indian context also illustrates complex tensions between individual rights and collective welfare, highlighting ethical dilemmas in areas such as reproductive healthcare, addiction treatment and vaccination policies.

Our findings underscore the need for culturally sensitive approaches to ethical decision-making. They also suggest that while international ethical codes provide a valuable framework, local cultural contexts must be considered to ensure ethical practices are both effective and respectful. Analized scientific literature highlights the ways in which ethical principles are understood and implemented in different countries. Further research that bridges global standards with local realities is needed.

## Data Availability

The original contributions presented in the study are included in the article/supplementary material, further inquiries can be directed to the corresponding author.

## Supplementary material

SUPPLEMENTARY TABLE 1 A summary of definitions and examples of issues related to the four main ethical principles based on a literature review.

## References

[1] Hofstede G Hofstede GJ Minkov M. Kultury i organizacje. 3rd. ed. Warszawa: PWN (2011).

[2] Bonecki M. Jerzy Kmita – interpretacja humanistyczna i społeczno-regulacyjna koncepcja kultury. Filoz Publiczna Eduk Demokrat. (2012) 1:178–98. doi: 10.14746/fped.2012.1.2.13

[3] Hajar R. The physician's oath: historical perspectives. Heart Views. (2017) 18:154–9. doi: 10.4103/HEARTVIEWS.HEARTVIEWS_131_17, PMID: 29326783 PMC5755201

[4] Delikat D Delikat T. Zdrowy dialog: Znajdź klucz do udanych relacji z pacjentem. 1st ed. Wrocław: MedPharm (2023).

[5] Pandya SK. History of medical ethics in India. Eubios J Asian Int Bioethics. (2000) 10:40–4.

[6] Pandya SK. Medical ethics in India: then and now. Acta Neurochir Suppl. (1999) 74:35–46. doi: 10.1007/978-3-7091-6387-0_8, PMID: 10549342

[7] Bendowska A Malak R Zok A Baum E. The ethics of translational audiology. Audiol Res. (2022) 12:273–80. doi: 10.3390/audiolres12030028, PMID: 35645198 PMC9149949

[8] Shetty N. Medical ethics and law. Indian J Orthop. (2023) 57:1744–7. doi: 10.1007/s43465-023-00972-w, PMID: 37881274 PMC10593724

[9] Dolma KG Das M Saravanabhavan SS . Investigation of an acute gastrointestinal illness outbreak linked to drinking water in a higher educational Institute in East Sikkim, India. Cureus. (2024) 16:e64050. doi: 10.7759/cureus.64050, PMID: 39114223 PMC11305448

[10] Bhagwat S Pai SA. Medical ethics in laboratory medicine: a review, with an oath for pathologists. Indian J Med Ethics. (2020) V:39–44. doi: 10.20529/IJME.2020.02, PMID: 32103817

[11] Bhojaraja MV Singhai P Sunil Kumar MM Sreelatha M. Withdrawal from Dialysis: why and when? Indian J Palliat Care. (2021) 27:S30–2. doi: 10.4103/ijpc.ijpc_66_21, PMID: 34188375 PMC8191749

[12] Math SB Manjunatha N Kumar CN Gowda GS Philip S Enara A . Sale of medicines by registered medical practitioners at their clinics: legal and ethical issues. Indian J Psychiatry. (2019) 61:786–S790. doi: 10.4103/psychiatry.IndianJPsychiatry_89_19, PMID: 31040475 PMC6482706

[13] Bhola P Sinha A Sonkar S Raguram A. Ethical dilemmas experienced by clinical psychology trainee therapists. Indian J Med Ethics. (2015) 12:206–12. doi: 10.20529/IJME.2015.055, PMID: 26322398

[14] Jain Y Kataria R. Rural blood availability: regulations must meet ethics. Indian J Med Ethics. (2016) 1:237–42. doi: 10.20529/IJME.2016.068, PMID: 27348617

[15] Sarkar MA Ozair A Singh KK Subash NR Bardhan M Khulbe Y. SARS-CoV-2 vaccination in India: considerations of hesitancy and bioethics in Global Health. Ann Glob Health. (2021) 87:124. doi: 10.5334/aogh.3530, PMID: 34963880 PMC8663743

[16] Sharma P Pardeshi G. COVID-19 vaccination in India: an ethical perspective. Diabetes Metab Syndr. (2021) 15:102314. doi: 10.1016/j.dsx.2021.102314, PMID: 34678577 PMC8516656

[17] John TJ Dharmapalan D. An ethical appraisal of the choice of vaccines against poliomyelitis. Indian J Med Ethics. (2019) IV:26–9. doi: 10.20529/IJME.2018.074, PMID: 30473498

[18] Basu S Garg S. Ethical clarity in clinical approaches to rabies prevention in re-exposure cases. Indian J Med Ethics. (2021) VI:1–4. doi: 10.20529/IJME.2020.091, PMID: 34081003

[19] Das NK Sil A. Evolution of ethics in clinical research and ethics committee. Indian J Dermatol. (2017) 62:373–9. doi: 10.4103/ijd.IJD_271_17, PMID: 28794547 PMC5527717

[20] Babu SS Varma RP. Disclosure of intimate partner violence while studying positive mental health in wheelchair users: ethical dilemmas. Indian J Med Ethics. (2023) 8:310–3. doi: 10.20529/IJME.2023.056, PMID: 38374673

[21] Janarthanan V Kumaran MS Nagrale NV Singh OG Raj KV. Legal and ethical issues associated with challenges in the implementation of the electronic medical record system and its current Laws in India. Cureus. (2024) 16:e56518. doi: 10.7759/cureus.56518, PMID: 38646271 PMC11026987

[22] Nimbalkar SM Patel DS. The medical termination of pregnancy act: need to keep pace with technology. Indian J Med Ethics. (2019) IV:59–64. doi: 10.20529/IJME.2018.096, PMID: 30916040

[23] Różyńska J. The ethical anatomy of payment for research participants. Med Health Care Philos. (2022) 25:449–64. doi: 10.1007/s11019-022-10092-1, PMID: 35610403 PMC9427899

[24] Pinto EP. The jurisprudence of emergency medical care in India: an ethics perspective. Indian J Med Ethics. (2017) 2:231–8. doi: 10.20529/IJME.2017.053, PMID: 28501791

[25] Singh AR Singh SA. Bioethical and other philosophical considerations in positive psychiatry. Mens Sana Monogr. (2016) 14:46–107. doi: 10.4103/0973-1229.193075, PMID: 28031624 PMC5179627

[26] Tibrewala M. Transgender persons and structural intersectionality: towards menstrual justice for all menstruators in India. *Indian*. J Med Ethics. (2024) IX:142–6. doi: 10.20529/IJME.2024.015, PMID: 38755765

[27] Basu S Sharma N. Under-recognised ethical dilemmas of diabetes care in resource-poor settings. Indian J Med Ethics. (2018) III:324–6. doi: 10.20529/IJME.2018.048 29981232

[28] Thiagesan R Gopichandran V Soundari H. Ethical framework to address barriers to healthcare for people with disabilities in India. Asian Bioeth Rev. (2023) 15:307–17. doi: 10.1007/s41649-023-00239-4, PMID: 36694541 PMC9853476

[29] Mukhopadhyay S Banerjee D. Physician assisted suicide in dementia: a critical review of global evidence and considerations from India. Asian J Psychiatr. (2021) 64:102802. doi: 10.1016/j.ajp.2021.102802, PMID: 34388669

[30] Gopichandran V. Moving from clinical to pragmatic equipoise in health policy and systems research. Indian J Med Ethics. (2020) V:1–6. doi: 10.20529/IJME.2020.77, PMID: 34018958

[31] Singh S. Ethical obstacles in health systems research in India: need for focused guidelines. Indian J Med Ethics. (2024) IX:31–4. doi: 10.20529/IJME.2023.062, PMID: 38375655

[32] Ajith KP Subramaniam S. COMMENT: ethics and professionalism of a community health worker: a virtue ethics approach. Indian J Med Ethics. (2022) VII:268–72. doi: 10.20529/IJME.2022.075, PMID: 36398394

[33] Kalra S Verma M. Justice, equality and liberty: inspiration from the Indian constitution for effective diabetes management. Indian J Med Ethics. (2021) 6:242. doi: 10.20529/IJME.2021.012 34018961

[34] Basu S Garg S. Antibiotic prescribing behavior among physicians: ethical challenges in resource-poor settings. J Med Ethics Hist Med. (2018) 11:5. PMID: 30258555 PMC6150921

[35] Gopichandran V. Ayushman Bharat National Health Protection Scheme: an ethical analysis. Asian Bioeth Rev. (2019) 11:69–80. doi: 10.1007/s41649-019-00083-5, PMID: 33717301 PMC7747303

[36] Dhikale PT Shrivastava SR Srinivasan S. Perspectives about professionalism among undergraduate students in a medical College in India: a qualitative study. Indian J Community Med. (2020) 45:230–4. doi: 10.4103/ijcm.IJCM_238_19, PMID: 32905183 PMC7467191

[37] Bari S Arora P Gupta AK Singh M Aggarwal AK. Tele-evidence: a videoconferencing tool as a viable alternative to physical appearance of doctors for the judicial summons. J Postgrad Med. (2018) 64:206–11. doi: 10.4103/jpgm.JPGM_243_17, PMID: 29943747 PMC6198697

[38] Arunachalam MA Halwai A. An analysis of the ethics of lockdown in India. Asian Bioeth Rev. (2020) 12:481–9. doi: 10.1007/s41649-020-00133-3, PMID: 32837557 PMC7347396

[39] Singh D Wadhwa R Daljit T. Human to humanoid: an evolving concept; issues and concerns of Neuroethics. Neurol India. (2022) 70:25–30. doi: 10.4103/0028-3886.338727, PMID: 35263849

[40] Srinivas G Maanasa R Meenakshi M Adaikalam JM Seshayyan S Muthuvel T. Ethical rationing of healthcare resources during COVID-19 outbreak: review. Ethics Med Public Health. (2021) 16:100633. doi: 10.1016/j.jemep.2021.100633, PMID: 33585668 PMC7869626

[41] Parmar A Patil V Sarkar S. Ethical management of substance use disorders: the Indian scenario. Indian J Med Ethics. (2017) 2:265–70. doi: 10.20529/IJME.2017.051, PMID: 28433964

[42] Nandimath OV Suhas S Malik Y Malathesh BC Math SB. National Medical Commission act, 2019 - the need for parity. Indian J Med Ethics. (2022) 7:229–30. doi: 10.20529/IJME.2022.030, PMID: 35699294

[43] Pitre A Bandewar SS. Law Commission of India report on the age of consent: denying justice and autonomy to adolescents. Indian J Med Ethics. (2024) IX:3–6. doi: 10.20529/IJME.2024.001, PMID: 38375643

[44] Baru RV Mohan M. Globalisation and neoliberalism as structural drivers of health inequities. Health Res Policy Syst. (2018) 16:91. doi: 10.1186/s12961-018-0365-2, PMID: 30301457 PMC6178247

[45] Bandewar SV Pitre A Lingam L. Five years post Nirbhaya: critical insights into the status of response to sexual assault. Indian J Med Ethics. (2018) 3:215–21. doi: 10.20529/IJME.2018.025, PMID: 29650498

[46] Madhiwalla N. Institutions should take responsibility for student suicides. Indian J Med Ethics. (2019) 4:252–3. doi: 10.20529/IJME.2019.029, PMID: 31213423

[47] Chańska W Grunt-Mejer K. The unethical use of ethical rhetoric: the case of flibanserin and pharmacologisation of female sexual desire. J Med Ethics. (2016) 42:701–4. doi: 10.1136/medethics-2016-103473, PMID: 27281797

[48] Zameska J. Why we should not "help bad choosers:" screening, nudging, and epistemic risk. Med Health Care Philos. (2024) 27:419–29. doi: 10.1007/s11019-024-10217-8, PMID: 38976145 PMC11310277

[49] Pietrzykowski T Smilowska K. The reality of informed consent: empirical studies on patient comprehension-systematic review. Trials. (2021) 22:57. doi: 10.1186/s13063-020-04969-w, PMID: 33446265 PMC7807905

[50] Olchowska-Kotala A Strządała A Barański J. Patients' values and desire for autonomy: an empirical study from Poland. J Bioeth Inq. (2023) 20:409–19. doi: 10.1007/s11673-023-10241-y, PMID: 36961586 PMC10624733

[51] Kroemeke A Sobczyk-Kruszelnicka M. Daily analysis of autonomy support and well-being in patient-caregiver dyads facing haematopoietic cell transplantation. Br J Health Psychol. (2022) 27:789–801. doi: 10.1111/bjhp.12573, PMID: 34806254

[52] Kroemeke A. Skala Postrzeganej Autonomii: struktura czynnikowa i właściwości psychometryczne polskiej adaptacji [Perceived Autonomy in Old Age scale: Factor structure and psychometric properties of the Polish adaptation]. Psychiatr Pol. (2015) 49:107–17. doi: 10.12740/PP/22616 25844414

[53] Domaradzki J Głodowska K Jabkowski P. Between autonomy and paternalism: attitudes of nursing personnel towards Jehovah's witnesses' refusal of blood transfusion. Int J Public Health. (2023) 68:1606291. doi: 10.3389/ijph.2023.1606291, PMID: 37600524 PMC10432684

[54] Patryn RK Zagaja A. Vaccinations-between free will and coercion. Hum Vaccin Immunother. (2016) 12:2204–5. doi: 10.1080/21645515.2016.1162936, PMID: 27070840 PMC4994740

[55] Zagaja A Patryn R Pawlikowski J Sak J. Informed consent in obligatory vaccinations? Med Sci Monit. (2018) 24:8506–9. doi: 10.12659/MSM.910393, PMID: 30472718 PMC6276839

[56] Pezdek K Dobrowolski R. The ethical code of conduct for physiotherapists-an axiological analysis. Int J Environ Res Public Health. (2023) 20:1362. doi: 10.3390/ijerph20021362, PMID: 36674118 PMC9859155

[57] Różyńska J Zawiła-Niedźwiecki J Maćkiewicz B Czarkowski M. Tough clinical decisions: experiences of polish physicians. HEC Forum. (2024) 36:111–30. doi: 10.1007/s10730-022-09491-x, PMID: 35939219 PMC10864525

[58] Domaradzki J. Patient rights, risk, and responsibilities in the genetic era - a right to know, a right not to know, or a duty to know? Ann Agric Environ Med. (2015) 22:156–62. doi: 10.5604/12321966.1141387, PMID: 25780847

[59] Domaradzki J Głodowska K Doron E Markwitz-Grzyb N Jabkowski P. Cultural competences among future nurses and midwives: a case of attitudes toward Jehovah's witnesses' stance on blood transfusion. BMC Med Educ. (2024) 24:663. doi: 10.1186/s12909-024-05646-1, PMID: 38879475 PMC11180393

[60] Kocańda K Głuszek S Szerla MK Domagała M. Respect for illiterate or unconscious patient's autonomy as a requirement for the legality of medical procedures in the polish healthcare system: a case report and review of the literature. Patient Saf Surg. (2022) 16:29. doi: 10.1186/s13037-022-00337-6, PMID: 36045394 PMC9429404

[61] Razmetaeva Y Sydorenko O. Euthanasia in the digital age: medical and legal issues and challenges. Georgian Med News. (2020) 298:175–80. 32141874

[62] Nykolyna KV. Reproductive choice: international ethical standards and prospects for legal regulation in certain European countries. Wiad Lek. (2020) 73:2056–61. doi: 10.36740/WLek202009230, PMID: 33148859

[63] Tiutiuhin VI Baida AO Bazeliuk VV. Legal restrictions on medical intervention during operation on female genitalia for non-medical purposes. Wiad Lek. (2020) 73:2909–14. doi: 10.36740/WLek202012234, PMID: 33611302

[64] Sulaieva O Dudin O Koshyk O Panko M Kobyliak N. Digital pathology implementation in cancer diagnostics: towards informed decision-making. Front Digit Health. (2024) 6:1358305. doi: 10.3389/fdgth.2024.1358305, PMID: 38873358 PMC11169727

[65] Mali S. Ethical considerations for surgeons. J Craniofac Surg. (2015) 26:6–9. doi: 10.1097/SCS.0000000000001292, PMID: 25569382

[66] Thaker SJ Figer BH Gogtay NJ Thatte UM. An audit of consent refusals in clinical research at a tertiary care center in India. J Postgrad Med. (2015) 61:257–63. doi: 10.4103/0022-3859.166515, PMID: 26440397 PMC4943370

[67] Gopichandran V Subramaniam S Palanisamy B Chidambaram P. Ethics and professionalism among community health workers in Tamil Nadu, India: a qualitative study. Dev World Bioeth. (2024) 24:151–66. doi: 10.1111/dewb.12414, PMID: 37462587

[68] Jain Y Phutke G. Issues in access to end-of-life care in low-resource areas. Indian J Med Ethics. (2018) 3:55–60. doi: 10.20529/IJME.2017.091, PMID: 29251606

[69] Ali F Gajera G Gowda GS Srinivasa P Gowda M. Consent in current psychiatric practice and research: an Indian perspective. Indian J Psychiatry. (2019) 61:667–S675. doi: 10.4103/psychiatry.IndianJPsychiatry_163_19, PMID: 31040455 PMC6482676

[70] Bandewar SV Chaudhuri L Duggal L Nagral S. The supreme court of India on euthanasia: too little, too late. Indian J Med Ethics. (2018) 3:91–4. doi: 10.20529/IJME.2018.028, PMID: 29724694

[71] Kishore RR. Aruna Shanbaug and the right to die with dignity: the battle continues. Indian J Med Ethics. (2016) 1:38–46. doi: 10.20529/IJME.2016.009, PMID: 26826660

[72] Biswas M. Living well till death. J Cancer Res Ther. (2015) 11:257–8. doi: 10.4103/0973-1482.159982, PMID: 26148579

[73] Timms O Pandya SK Jesani A Srinivasan S. Centring patient autonomy in DNAR decisions. *Indian*. J Med Ethics. (2020) 5:340–1. doi: 10.20529/IJME.2020.096, PMID: 34018951

[74] Kale B Jaiswal P Masurkar D. Living will: today’s thoughts and actions. J Family Med Prim Care. (2024) 13:20–3. doi: 10.4103/jfmpc.jfmpc_482_22, PMID: 38482288 PMC10931894

[75] Ghooi RB Dhru K Jaywant S. The urgent need for advance directives in India. Indian J Med Ethics. (2016) 1:242–9. doi: 10.20529/IJME.2016.069, PMID: 27731296

[76] Arunachaleeswaran P Bhan A. COMMENT: age and autonomy: an ethical dilemma in community mental health. Indian J Med Ethics. (2022) VII:286–90. doi: 10.20529/IJME.2022.076, PMID: 36398393

[77] Remien K Kanchan T. Parental Consent. Treasure Island. FL: StatPearls Publishing (2022). 32310349

[78] Jain D Rastogi A. RESEARCH ARTICLE: adolescent abortions in the Covid-19 landscape: exposing the legal Achilles' heel. Indian J Med Ethics. (2024) 9:48–57. doi: 10.20529/IJME.2024.002, PMID: 38375642

[79] Nadkarni A Kapoor A Pathare S. COVID-19 and forced alcohol abstinence in India: the dilemmas around ethics and rights. Int J Law Psychiatry. (2020) 71:101579. doi: 10.1016/j.ijlp.2020.101579, PMID: 32768113 PMC7237931

[80] Agrawal S Agarwal A Jain Y. Convalescent plasma, political narrative, and public health ethics in the Covid-19 pandemic. Indian J Med Ethics. (2021) 6:226–8. doi: 10.20529/IJME.2021.029, PMID: 34287194

[81] Srinivasan S. The vaccine mandates judgment: some reflections. Indian J Med Ethics. (2023) VIII:134–40. doi: 10.20529/IJME.2022.056, PMID: 36880474

[82] Rajagopal MR. To comfort always: are we ignoring this duty in Covid protocols? Indian J Med Ethics. (2020) 5:189–91. doi: 10.20529/IJME.2020.071, PMID: 33295287

[83] Kurpad AV Ghosh S Thomas T Bandyopadhyay S Goswami R Gupta A . Perspective: when the cure might become the malady: the layering of multiple interventions with mandatory micronutrient fortification of foods in India. Am J Clin Nutr. (2021) 114:1261–6. doi: 10.1093/ajcn/nqab245, PMID: 34320172

[84] Ansari H Yeravdekar R. Respectful maternity care: a national landscape review. Natl Med J India. (2019) 32:290–3. doi: 10.4103/0970-258X.295957, PMID: 32985445

[85] Mondal D Karmakar S Banerjee A. Women's autonomy and utilization of maternal healthcare in India: evidence from a recent national survey. PLoS One. (2020) 15:e0243553. doi: 10.1371/journal.pone.0243553, PMID: 33296428 PMC7725295

[86] Kashyap GC Govind B Srivastava S. A true face of Indian married couples: effect of age and education on control over own sexuality and sexual violence. PLoS One. (2021) 16:e0254005. doi: 10.1371/journal.pone.0254005, PMID: 34288932 PMC8294513

[87] Patel SK Saggurti N Pachauri S Prabhakar P. Correlates of mental depression among female sex workers in southern India. Asia Pac J Public Health. (2015) 27:809–19. doi: 10.1177/1010539515601480, PMID: 26307144

[88] Jindal UN. Mid-life fertility: challenges & policy planning. Indian J Med Res. (2018) 148:S15–26. doi: 10.4103/ijmr.IJMR_647_18, PMID: 30964078 PMC6469367

[89] Goswami GK. The genetic truth of surrogate parentage. Med Leg J. (2015) 83:188–93. doi: 10.1177/0025817215576877, PMID: 25782415

[90] Patel T. Experiencing abortion rights in India through issues of autonomy and legality: a few controversies. Glob Public Health. (2018) 13:702–10. doi: 10.1080/17441692.2018.1424920 29343181

[91] Gupta M Iyengar K Singla N Kaur K Verma M Singla R . Rights-based reproductive services in medical schools in Rajasthan, Gujarat and Chandigarh, India: baseline findings of mixed-methods implementation research. Contracept Reprod Med. (2024) 9:58. doi: 10.1186/s40834-024-00316-5, PMID: 39543693 PMC11566405

[92] Majumder K Sarkar M Mallick R Mondal S Chouhan P. Does women's decision-making autonomy matter in utilization of antenatal care services in India? An analysis from a nationally representative survey. PLoS One. (2024) 19:e0308576. doi: 10.1371/journal.pone.0308576, PMID: 39172924 PMC11340970

[93] Barua A Rastogi A Deepa V Jain D Gupte M Mallik R . The MTP 2020 amendment bill: anti-rights subjectivity. Sex Reprod Health Matters. (2020) 28:1795447. doi: 10.1080/26410397.2020.1795447, PMID: 32720587 PMC7888022

[94] Mitra P. Invisible women in reproductive technologies: critical reflections. Indian J Med Ethics. (2018) 3:113–9. doi: 10.20529/IJME.2018.031, PMID: 29724696

[95] Sil A Das NK. Informed consent process: Foundation of the Researcher-participant Bond. Indian J Dermatol. (2017) 62:380–6. doi: 10.4103/ijd.IJD_272_17, PMID: 28794548 PMC5527718

[96] Figer BH Lamture SS Gandhi T Chauhan A Gvalani A Gogtay NJ . A survey of knowledge and variables influencing perceptions about clinical research: a cross-sectional study from Mumbai. Perspect Clin Res. (2021) 12:93–9. doi: 10.4103/picr.PICR_97_19, PMID: 34012906 PMC8112328

[97] Kadam RA. Informed consent process: a step further towards making it meaningful! Perspect Clin Res. (2017) 8:107–12. doi: 10.4103/picr.PICR_147_16, PMID: 28828304 PMC5543760

[98] Subramani S. Patient autonomy within real or valid consent: Samira Kohli's case. Indian J Med Ethics. (2017) 2:184–9. doi: 10.20529/IJME.2017.038, PMID: 28195536

[99] Patil A Chawathey S Malim A. Adequacy of informed consent in elective surgical procedures: a study in a Navi Mumbai tertiary care Centre. Cureus. (2023) 15:e41777. doi: 10.7759/cureus.41777 37449289 PMC10337701

[100] Aneja J Arora S. Pregnancy and severe mental illness: confounding ethical doctrines. *Indian*. J Med Ethics. (2020) V(2):133-139:133–9. doi: 10.20529/IJME.2020.037, PMID: 32393450

[101] Vishwanathan K Nimbalkar S. Cosmetic limb lengthening in a patient of normal stature: ethical considerations. Indian J Med Ethics. (2017) 2:45–8. doi: 10.20529/IJME.2017.009, PMID: 27866145

[102] Vajawat B Hegde PR Malathesh BC Kumar CN Sivakumar PT Math SB. Palliative care and legal issues in geriatric psychiatry. Indian J Psychol Med. (2021) 43:S31–6. doi: 10.1177/02537176211031077, PMID: 34732952 PMC8543619

[103] Mukhopadhyay D Choudhari SG. Clinical reasoning skills among second-phase medical students in West Bengal, India: an exploratory study. Cureus. (2024) 16:e68839. doi: 10.7759/cureus.68839, PMID: 39376810 PMC11456746

[104] Chowdhury S Vavachan RT Empathy N. Moral sensitivity, and prosocial behavior among medical undergraduates in a south Indian tertiary care teaching institute: an analytical cross-sectional study. Cureus. (2024) 16:e70392. doi: 10.7759/cureus.70392, PMID: 39469398 PMC11515942

[105] Chandra A. Ethical issues and proposed solutions in conducting practical assessment of medical students involving patients. Indian J Med Ethics. (2024) 9:217–22. doi: 10.20529/IJME.2024.034 39183612

[106] Ahmed HS. Beyond traditional tools: exploring convolutional neural networks as innovative prognostic models in pancreatic ductal adenocarcinoma. Arq Gastroenterol. (2024) 61:e23107. doi: 10.1590/S0004-2803.24612023-117 38511794

[107] Banerjee A Sarangi PK Kumar S. Medical doctors' perceptions of artificial intelligence (AI) in healthcare. Cureus. (2024) 16:e70508. doi: 10.7759/cureus.70508, PMID: 39479138 PMC11524062

[108] Chuented P Puranitee P Pakakasama S Meepanya S. Factors affecting residents' internal motivation, grit, and well-being. BMC Med Educ. (2023) 23:779. doi: 10.1186/s12909-023-04679-2, PMID: 37858074 PMC10588185

[109] Manjavong M Srinonprasert V Limpawattana P Chindaprasirt J Pairojkul S Kuichanuan T . Comparison of Thai older patients' wishes and nurses' perceptions regarding end-of-life care. Nurs Ethics. (2019) 26:2006–15. doi: 10.1177/0969733019826410, PMID: 30841782

[110] Ho R Chantagul N. Support for voluntary and nonvoluntary euthanasia: what roles do conditions of suffering and the identity of the terminally ill play? Omega. (2015) 70:251–77. doi: 10.1177/0030222815568958, PMID: 26036055

[111] Phanuphak N Seekaew P Phanuphak P. Optimising treatment in the test-and-treat strategy: what are we waiting for? Lancet HIV. (2019) 6:e715–22. doi: 10.1016/S2352-3018(19)30236-X 31515166

[112] Różyńska J. Taking the principle of the primacy of the human being seriously. Med Health Care Philos. (2021) 24:547–62. doi: 10.1007/s11019-021-10043-2, PMID: 34318429 PMC8557179

[113] Panaso C. Working experience of nurse anesthetists with beneficence for patients. Nurs Ethics. (2024) 31:508–20. doi: 10.1177/09697330231197706, PMID: 38165281

[114] Doraiswamy V Natarajan L Venkatesh CT. Supraventricular tachycardia in one of the twins: the ethical dilemmas involved in treatment. Ann Pediatr Cardiol. (2020) 13:150–2. doi: 10.4103/apc.APC_204_19, PMID: 32641889 PMC7331848

[115] Ghoshal R. The social value of research: interrogating the paradoxes. Indian J Med Ethics. (2018) 3:9–15. doi: 10.20529/IJME.2018.001, PMID: 29477983

[116] Sil A Das NK. Ethics of safety reporting of a clinical trial. Indian J Dermatol. (2017) 62:387–91. doi: 10.4103/ijd.IJD_273_17, PMID: 28794549 PMC5527719

[117] Rechel B Maresso A Sagan A. The Ethics of Health Policy. Copenhagen: European Observatory on Health Systems and Policies (2018). 29897704

[118] LeDoux J Mann C Demoratz M Young J. Addressing spiritual and religious influences in care delivery. Prof Case Manag. (2019) 24:142–7. doi: 10.1097/NCM.0000000000000346, PMID: 30946252

[119] Mona LR Cameron RP Clemency CC. Disability culturally competent sexual healthcare. Am Psychol. (2017) 72:1000–10. doi: 10.1037/amp0000283, PMID: 29283660

[120] Castaneda-Guarderas A Glassberg J Grudzen CR Ngai KM Samuels-Kalow ME Shelton E . Shared decision making with vulnerable populations in the emergency department. Acad Emerg Med. (2016) 23:1410–6. doi: 10.1111/acem.13134, PMID: 27860022 PMC9165457

[121] Turner L. Medical ethics in a multicultural society. J R Soc Med. (2001) 94:592–4. doi: 10.1177/014107680109401114, PMID: 11691903 PMC1282251

