# Supplementary material for: Ethical principles across countries: does ‘ethical’ mean the same everywhere?
Source: Front Public Health. 2025 Jun 11;13:1579778. doi: 10.3389/fpubh.2025.1579778 (PMC12188308; doi:10.3389/fpubh.2025.1579778)
Supplement: SUPPLEMENTARY TABLE 1 — A summary of definitions and examples of issues related to the four main ethical principles based on a literature review. [file Data_Sheet_1.pdf]

	Poland	Ukraine	India	Thailand
**Non-maleficence**	"Balancing the risk and benefits of treatment"<br>-medical research<br><br>*Bibliography*<br>*Bendowska A, Malak R, Zok A, Baum E. The Ethics of Translational Audiology. Audiol Res. 2022;12(3):273-280. Published 2022 May 13. (22)*	no data	"Prioritizing harm prevention"<br>-medical errors<br>-overdiagnosis<br>-financial burdens<br>-informed decision-making<br>-vaccine safety<br>-clinical research<br>-abortion<br><br>*Bibliography*<br>*Shetty N. Medical Ethics and Law. Indian J Orthop. 2023;57(11):1744-1747. Published 2023 Sep 4. (7)*<br>*Dolma KG, Das M, Saravanabhavan SS, et al. Investigation of an Acute Gastrointestinal Illness Outbreak Linked to Drinking Water in a Higher Educational Institute in East Sikkim, India. Cureus. 2024;16(7):e64050. Published 2024 Jul 7. (8)*<br>*Bhagwat S, Pai SA. Medical ethics in laboratory medicine: A review, with an oath for pathologists. Indian J Med Ethics. 2020;V(1):39-44. (9)*<br>*Bhojaraja MV, Singhai P, Sunil Kumar MM, Sreelatha M. Withdrawal from Dialysis: Why and When? Indian J Palliat Care. 2021;27(Suppl 1):S30-S32. (10)*<br>*Math SB, Manjunatha N, Kumar CN, et al. Sale of medicines by Registered Medical Practitioners at their clinics: Legal and*	no data

			ethical issues. Indian J Psychiatry. 2019;61(Suppl 4):S786-S790. (11) Bhola P, Sinha A, Sonkar S, Raguram A. Ethical dilemmas experienced by clinical psychology trainee therapists. Indian J Med Ethics. 2015;12(4):206-212. (12)<br>*Jain Y, Kataria R. Rural blood availability: regulations must meet ethics. Indian J Med Ethics. 2016;1(4):237-242. (13)*<br>*Sarkar MA, Ozair A, Singh KK, Subash NR, Bardhan M, Khulbe Y. SARS-CoV-2 Vaccination in India: Considerations of Hesitancy and Bioethics in Global Health. Ann Glob Health. 2021;87(1):124. Published 2021 Dec 10. (14)*<br>*Sharma P, Pardeshi G. COVID-19 vaccination in India: An ethical perspective. Diabetes Metab Syndr. 2021;15(6):102314. (15)*<br>*John TJ, Dharmapalan D. An ethical appraisal of the choice of vaccines against Poliomyelitis. Indian J Med Ethics. 2019;4(1):26-29. (16)*<br>*Basu S, Garg S. Ethical clarity in clinical approaches to rabies prevention in re-exposure cases. Indian J Med Ethics. 2021;VI(1):1-4. (17)*	

			Das NK, Sil A. Evolution of Ethics in Clinical Research and Ethics Committee. Indian J Dermatol. 2017;62(4):373-379. (18)<br>*Babu SS, Varma RP. Disclosure of intimate partner violence while studying positive mental health in wheelchair users: ethical dilemmas. Indian J Med Ethics. 2023;VIII(4):310-313. (19)*<br>*Janarthanan V, Kumaran MS, Nagrale NV, Singh OG, Raj KV. Legal and Ethical Issues Associated With Challenges in the Implementation of the Electronic Medical Record System and Its Current Laws in India. Cureus. 2024;16(3):e56518. Published 2024 Mar 20. (20)*<br>*Nimbalkar SM, Patel DS. The Medical Termination of Pregnancy Act: Need to keep pace with technology. Indian J Med Ethics. 2019;4(1):59-64. (21)*	
**Justice**	"Equitable access to healthcare benefits and fair distribution of burdens"<br>-access to modern technology<br>-communication with patients<br>- clinical research<br><br>*Bibliography*<br>*Bendowska A, Malak R, Zok A, Baum E. The Ethics of Translational*	no data	"Equal respect and fair treatment"<br>-systemic barriers<br>-equitable access to resources<br>-clinical research<br>-vaccination<br>-digital health<br>-legal frameworks<br><br>*Bibliography*<br>*Pinto EP. The jurisprudence of emergency medical care in India: an*	no data

	Audiology. Audiol Res. 2022;12(3):273-280. Published 2022 May 13. (22)<br>*Różyńska J. The ethical anatomy of payment for research participants. Med Health Care Philos. 2022;25(3):449-464. (23)*		*ethics perspective. Indian J Med Ethics. 2017;2(4):231-238. (24)*<br>*Singh AR, Singh SA. Bioethical and Other Philosophical Considerations in Positive Psychiatry. Mens Sana Monogr. 2016;14(1):46-107. (25)*<br>*Tibrewala M. Transgender persons and structural intersectionality: Towards menstrual justice for all menstruators in India. Indian J Med Ethics. 2024;IX(2):142-146. (26)*<br>*Basu S, Sharma N. Under-recognised ethical dilemmas of diabetes care in resource-poor settings. Indian J Med Ethics. 2018;3(4):324-326. (27)*<br>*Thiagesan R, Gopichandran V, Soundari H. Ethical Framework to Address Barriers to Healthcare for People with Disabilities in India. Asian Bioeth Rev. Published online January 20, 2023. (28)*<br>*Mukhopadhyay S, Banerjee D. Physician assisted suicide in dementia: A critical review of global evidence and considerations from India. Asian J Psychiatr. 2021;64:102802. (29)*<br>*Gopichandran V. Moving from clinical to pragmatic equipoise in health*	

			policy and systems research. Indian J Med Ethics. 2020;V(4):1-6. (30) Singh S. Ethical obstacles in health systems research in India: Need for focused guidelines. Indian J Med Ethics. 2024;IX(1):31-34. (31) Ajith KP, Subramaniam S. COMMENT: Ethics and professionalism of a community health worker: A virtue ethics approach. Indian J Med Ethics. 2022;VII(4):268-272. (32) Kalra S, Verma M. Justice, equality and liberty: Inspiration from the Indian Constitution for effective diabetes management. Indian J Med Ethics. Published online February 23, 2021. (33) Basu S, Garg S. Antibiotic prescribing behavior among physicians: ethical challenges in resource-poor settings. J Med Ethics Hist Med. 2018;11:5. Published 2018 May 12. (34) Gopichandran V. Ayushman Bharat National Health Protection Scheme: an Ethical Analysis. Asian Bioeth Rev. 2019;11(1):69-80. Published 2019 Apr 3. (35) Dhikale PT, Shrivastava SR, Srinivasan S. Perspectives about Professionalism among	

			Undergraduate Students in a Medical College in India: A Qualitative Study. Indian J Community Med. 2020;45(2):230-234. *(36)* *Bari S, Arora P, Gupta AK, Singh M, Aggarwal AK. Tele-evidence: A videoconferencing tool as a viable alternative to physical appearance of doctors for the judicial summons. J Postgrad Med. 2018;64(4):206-211.* *(37)* *Arunachalam MA, Halwai A. An analysis of the ethics of lockdown in India. Asian Bioeth Rev. 2020;12(4):481-489. Published 2020 Jul 9.* *(38)* *Singh D, Wadhwa R, Daljit T. Human to Humanoid: An Evolving Concept; Issues and Concerns of Neuroethics. Neurol India. 2022;70(1):25-30.* *(39)* *Srinivas G, Maanasa R, Meenakshi M, Adaikalam JM, Seshayyan S, Muthuvel T. Ethical rationing of healthcare resources during COVID-19 outbreak: Review. Ethics Med Public Health. 2021;16:100633.* *(40)* *Parmar A, Patil V, Sarkar S. Ethical management of substance use disorders: the Indian scenario. Indian J Med Ethics. 2017;2(4):265-270.*	

			(41) Nandimath OV, Suhas S, Malik Y, Malathesh BC, Math SB. National Medical Commission Act, 2019 - the need for parity. Indian J Med Ethics. 2022;VII(3):229-230. (42) Pitre A, Bandewar SS. Law Commission of India report on the age of consent: Denying justice and autonomy to adolescents. Indian J Med Ethics. 2024;IX(1):3-6. (43) Baru RV, Mohan M. Globalisation and neoliberalism as structural drivers of health inequities. Health Res Policy Syst. 2018;16(Suppl 1):91. Published 2018 Oct 9. (44) Pai SN, Chandra KS. Medical Termination of Pregnancy Act of India: Treading the Path between Practical and Ethical Reproductive Justice. Indian J Community Med. 2023;48(4):510-513. (45) Bandewar SV, Pitre A, Lingam L. Five years post Nirbhaya: Critical insights into the status of response to sexual assault. Indian J Med Ethics. 2018;3(3):215-221. (46) Madhiwalla N. Institutions should take responsibility for student suicides. Indian J Med Ethics. 2019;4(3):252. (47)	
**Autonomy**	“Ability to make informed choices” -informed consent	“Respecting individual autonomy in	“Right to self-determination and informed	“Autonomy is not only theoretical

	-clear communication<br>-respect for individual decision-making<br>-children’s autonomy<br>-vaccination<br>-clinical research<br>-refusing medical information<br>-healthcare professional's autonomy<br>-religious beliefs<br><br>*Bibliography*<br>*Chańska W, Grunt-Mejer K. The unethical use of ethical rhetoric: the case of flibanserin and pharmacologisation of female sexual desire. J Med Ethics. 2016;42(11):701-704. (48)*<br>*Zameska J. Why we should not "help bad choosers:" screening, nudging, and epistemic risk. Med Health Care Philos. 2024;27(3):419-429. (49)*<br>*Pietrzykowski T, Smilowska K. The reality of informed consent: empirical studies on patient comprehension-systematic review. Trials. 2021;22(1):57. Published 2021 Jan 14. (50)*<br>*Olchowska-Kotala A, Strządała A, Barański J. Patients' Values and Desire for Autonomy: An Empirical Study from Poland. J Bioeth Inq. 2023;20(3):409-419. (51)*<br>*Kroemeke A, Sobczyk-Kruszelnicka M. Daily analysis of autonomy support and well-being in*	decision-making<br>-euthanasia<br>-reproductive rights<br>-cultural influence<br>-AI<br><br>*Bibliography*<br>*Razmetaeva Y, Sydorenko O. Euthanasia in the digital age: medical and legal issues and challenges. Georgian Med News. 2020;(298):175-180. (62)*<br>*Nykolyna KV. Reproductive choice: International ethical standards and prospects for legal regulation in certain European countries. Wiad Lek. 2020;73(9 cz. 2):2056-2061. (63)*<br>*Tiutiuhin VI, Baida AO, Bazeliuk VV. Legal restrictions on medical intervention during operation on female genitalia for non-medical purposes. Wiad Lek. 2020;73(12 cz 2):2909-2914. (64)*<br>*Sulaieva O, Dudin O, Koshyk O, Panko M, Kobyliak N. Digital pathology implementation in cancer diagnostics: towards informed decision-making. Front Digit Health. 2024;6:1358305. Published 2024 May 30. (65)*	decision-making”<br>-traditional social structures<br>-autonomy of medical workers<br>-children’s autonomy<br>-public health vs personal autonomy<br>-women’s autonomy<br>-clinical research<br>-new technologies<br><br>*Bibliography*<br>*Mali S. Ethical considerations for surgeons. J Craniofac Surg. 2015;26(1):6-9. (66)*<br>*Thaker SJ, Figer BH, Gogtay NJ, Thatte UM. An audit of consent refusals in clinical research at a tertiary care center in India. J Postgrad Med. 2015;61(4):257-263. (67)*<br>*Gopichandran V, Subramaniam S, Palanisamy B, Chidambaram P. Ethics and professionalism among community health workers in Tamil Nadu, India: A qualitative study. Dev World Bioeth. 2024;24(3):151-166. (68)*<br>*Jain Y, Phutke G. Issues in access to end-of-life care in low-resource areas. Indian J Med Ethics. 2018;3(1):55-60. (69)*<br>*Ali F, Gajera G, Gowda GS, Srinivasa P, Gowda M. Consent in current psychiatric practice and research: An Indian perspective. Indian J Psychiatry. 2019;61(Suppl 4):S667-S675. (70)*<br>*Bandewar SV,*	concept but it is also linked to personal lifestyle factors”<br>-factors influencing health workers autonomy end-of-life care<br>-euthanasia<br>-HIV treatment<br><br>*Bibliography*<br>*Chuented P, Puranitee P, Pakakasama S, Meepanya S. Factors affecting residents' internal motivation, grit, and well-being. BMC Med Educ. 2023;23(1):779. Published 2023 Oct 19. (109)*<br>*Manjavong M, Srinonprasert V, Limpawattana P, et al. Comparison of Thai older patients' wishes and nurses' perceptions regarding end-of-life care. Nurs Ethics. 2019;26(7-8):2006-2015. (110)*<br>*Ho R, Chantagul N. Support for voluntary and nonvoluntary euthanasia: what roles do conditions of suffering and the identity of the terminally ill play?. Omega (Westport). 2015;70(3):251-277. (111)*<br>*Phanuphak N, Seekaew P, Phanuphak P. Optimising treatment in the*

	patient-caregiver dyads facing haematopoietic cell transplantation. Br J Health Psychol. 2022;27(3):789-801. (52)<br>*Kroemeke A. Skala Postrzeganej Autonomii: struktura czynnikowa i właściwości psychometryczne polskiej adaptacji [Perceived Autonomy in Old Age scale: Factor structure and psychometric properties of the Polish adaptation]. Psychiatr Pol. 2015;49(1):107-117. (53)*<br>*Domaradzki J, Głodowska K, Jabkowski P. Between Autonomy and Paternalism: Attitudes of Nursing Personnel Towards Jehovah's Witnesses' Refusal of Blood Transfusion. Int J Public Health. 2023;68:1606291. Published 2023 Aug 3. (54)*<br>*Patryn RK, Zagaja A. Vaccinations-Between free will and coercion. Hum Vaccin Immunother. 2016;12(8):2204-2205. (55)*<br>*Zagaja A, Patryn R, Pawlikowski J, Sak J. Informed Consent in Obligatory Vaccinations?. Med Sci Monit. 2018;24:8506-8509. Published 2018 Nov 25. (56)*<br>*Pezdek K, Dobrowolski R. The Ethical Code of Conduct for Physiotherapists—An Axiological Analysis.*		*Chaudhuri L, Duggal L, Nagral S. The Supreme Court of India on euthanasia: Too little, too late. Indian J Med Ethics. 2018;3(2):91-94. (71)*<br>*Kishore RR. Aruna Shanbaug and the right to die with dignity: the battle continues. Indian J Med Ethics. 2016;1(1):38-46. (72)*<br>*Biswas M. Living well till death. J Cancer Res Ther. 2015;11(2):257-258. (73)*<br>*Timms O, Pandya SK, Jesani A, Srinivasan S. Centring patient autonomy in DNAR decisions. Indian J Med Ethics. 2020;V(4):1-3. (74)*<br>*Kale B, Jaiswal P, Masurkar D. Living will: Today's thoughts and actions. J Family Med Prim Care. 2024;13(1):20-23. (75)*<br>*Ghooi RB, Dhru K, Jaywant S. The urgent need for advance directives in India. Indian J Med Ethics. 2016;1(4):242-249. (76)*<br>*Arunachaleeswaran P, Bhan A. COMMENT: Age and autonomy: An ethical dilemma in community mental health. Indian J Med Ethics. 2022;VII(4):286-290. (77)*<br>*Remien K, Kanchan T. Parental Consent. In: StatPearls. Treasure Island (FL):*	*test-and-treat strategy: what are we waiting for?. Lancet HIV. 2019;6(10):e715-e722. (112)*

	Int J Environ Res Public Health. 2023;20(2):1362. Published 2023 Jan 12. (57)<br>*Różyńska J, Zawiła-Niedźwiecki J, Maćkiewicz B, Czarkowski M. Tough Clinical Decisions: Experiences of Polish Physicians. HEC Forum. 2024;36(1):111-130. (58)*<br>*Domaradzki J. Patient rights, risk, and responsibilities in the genetic era—a right to know, a right not to know, or a duty to know?. Ann Agric Environ Med. 2015;22(1):156-162. (59)*<br>*Domaradzki J, Głodowska K, Doron E, Markwitz-Grzyb N, Jabkowski P. Cultural competences among future nurses and midwives: a case of attitudes toward Jehovah's Witnesses' stance on blood transfusion. BMC Med Educ. 2024;24(1):663. Published 2024 Jun 15. (60)*<br>*Kocańda K, Głuszek S, Szerla MK, Domagała M. Respect for illiterate or unconscious patient's autonomy as a requirement for the legality of medical procedures in the Polish healthcare system: a case report and review of the literature. Patient Saf Surg. 2022;16(1):29. Published 2022 Aug 31. (61)*		*StatPearls Publishing; September 18, 2022. (78)*<br>*Jain D, Rastogi A. RESEARCH ARTICLE: Adolescent abortions in the Covid-19 landscape: Exposing the legal Achilles' heel. Indian J Med Ethics. 2024;IX(1):48-57. (79)*<br>*Nadkarni A, Kapoor A, Pathare S. COVID-19 and forced alcohol abstinence in India: The dilemmas around ethics and rights. Int J Law Psychiatry. 2020;71:101579. (80)*<br>*Agrawal S, Agarwal A, Jain Y. Convalescent plasma, political narrative, and public health ethics in the Covid-19 pandemic. Indian J Med Ethics. 2021;VI(3):1-7. (81)*<br>*Srinivasan S. The vaccine mandates judgment: Some reflections. Indian J Med Ethics. 2023;VIII(2):134-140 . (82)*<br>*Rajagopal MR. To comfort always: Are we ignoring this duty in Covid protocols?. Indian J Med Ethics. 2020;V(3):189-191. (83)*<br>*Kurpad AV, Ghosh S, Thomas T, et al. Perspective: When the cure might become the malady: the layering of multiple interventions with mandatory micronutrient fortification of foods in India. Am J Clin*	

	Bendowska A, Malak R, Zok A, Baum E. The Ethics of Translational Audiology. Audiol Res. 2022;12(3):273-280. Published 2022 May 13. (22)<br>*Różyńska J. The ethical anatomy of payment for research participants. Med Health Care Philos. 2022;25(3):449-464. (23)*		*Nutr. 2021;114(4):1261-1266. (84)*<br>*Ansari H, Yeravdekar R. Respectful maternity care: A national landscape review. Natl Med J India. 2019;32(5):290-293. (85)*<br>*Mondal D, Karmakar S, Banerjee A. Women's autonomy and utilization of maternal healthcare in India: Evidence from a recent national survey. PLoS One. 2020;15(12):e0243553. (86)*<br>*Kashyap GC, Govind B, Srivastava S, R V, Bango M, Shaw S. A true face of Indian married couples: Effect of age and education on control over own sexuality and sexual violence. PLoS One. 2021;16(7):e0254005. (87)*<br>*Patel SK, Saggurti N, Pachauri S, Prabhakar P. Correlates of mental depression among female sex workers in southern India. Asia Pac J Public Health. 2015;27(8):809-819. (88)*<br>*Jindal UN. Mid-life fertility: Challenges & policy planning. Indian J Med Res. 2018;148(Suppl):S15-S26. (89)*<br>*Goswami GK. The genetic truth of surrogate parentage. Med Leg J. 2015;83(4):188-193. (90)*<br>*Patel T. Experiencing abortion rights in India through issues*	

			of autonomy and legality: A few controversies. Glob Public Health. 2018;13(6):702-710. (91)<br>*Gupta M, Iyengar K, Singla N, et al. Rights-based reproductive services in medical schools in Rajasthan, Gujarat and Chandigarh, India: Baseline findings of mixed-methods implementation research. Contracept Reprod Med. 2024;9(1):58. (92)*<br>*Majumder K, Sarkar M, Mallick R, Mondal S, Chouhan P. Does women's decision-making autonomy matter in utilization of antenatal care services in India? An analysis from a nationally representative survey. PLoS One. 2024;19(8):e0308576. (93)*<br>*Barua A, Rastogi A, Deepa V, et al. The MTP 2020 Amendment Bill: anti-rights subjectivity. Sex Reprod Health Matters. 2020;28(1):1795447. (94)*<br>*Mitra P. Invisible women in reproductive technologies: Critical reflections. Indian J Med Ethics. 2018;3(2):113-119. (95)*<br>*Sil A, Das NK. Informed Consent Process: Foundation of the Researcher-participant Bond. Indian J*	

			Dermatol. 2017;62(4):380-386. (96)<br>*Figer BH, Lamture SS, Gandhi T, et al. A survey of knowledge and variables influencing perceptions about clinical research: A cross-sectional study from Mumbai. Perspect Clin Res. 2021;12(2):93-99. (97)*<br>*Kadam RA. Informed consent process: A step further towards making it meaningful!. Perspect Clin Res. 2017;8(3):107-112. (98)*<br>*Subramani S. Patient autonomy within real or valid consent: Samira Kohli's case. Indian J Med Ethics. 2017;2(3):184-189. (99)*<br>*Patil A, Chawathey S, Malim A. Adequacy of Informed Consent in Elective Surgical Procedures: A Study in a Navi Mumbai Tertiary Care Centre. Cureus. 2023;15(7):e41777. (100)*<br>*Aneja J, Arora S. Pregnancy and severe mental illness: Confounding ethical doctrines. Indian J Med Ethics. 2020;V(2):133-139. (101)*<br>*Vishwanathan K, Nimbalkar S. Cosmetic limb lengthening in a patient of normal stature: ethical considerations. Indian J Med Ethics. 2017;2(1):45-48. (102)*	

			Vajawat B, Hegde PR, Malathesh BC, Kumar CN, Sivakumar PT, Math SB. Palliative Care and Legal Issues in Geriatric Psychiatry. Indian J Psychol Med. 2021;43(5 Suppl):S31-S36. (103)<br>*Mukhopadhyay D, Choudhari SG. Clinical Reasoning Skills Among Second-Phase Medical Students in West Bengal, India: An Exploratory Study. Cureus. 2024;16(9):e68839. (104)*<br>*Chowdhury S, K G N, Vavachan RT. Empathy, Moral Sensitivity, and Prosocial Behavior Among Medical Undergraduates in a South Indian Tertiary Care Teaching Institute: An Analytical Cross-Sectional Study. Cureus. 2024;16(9):e70392. (105)*<br>*Chandra A. Ethical issues and proposed solutions in conducting practical assessment of medical students involving patients. Indian J Med Ethics. 2024;IX(3):217-222. (106)*<br>*Ahmed HS. Beyond traditional tools: Exploring convolutional neural networks as innovative prognostic models in pancreatic ductal adenocarcinoma. Arq Gastroenterol. 2024;61:e23107. (107)*	

			Banerjee A, Sarangi PK, Kumar S. Medical Doctors' Perceptions of Artificial Intelligence (AI) in Healthcare. Cureus. 2024;16(9):e70508. (108)<br>*Bhagwat S, Pai SA. Medical ethics in laboratory medicine: A review, with an oath for pathologists. Indian J Med Ethics. 2020;V(1):39-44.(9)*<br>*Math SB, Manjunatha N, Kumar CN, et al. Sale of medicines by Registered Medical Practitioners at their clinics: Legal and ethical issues. Indian J Psychiatry. 2019;61(Suppl 4):S786-S790. (11)*<br>*Bhola P, Sinha A, Sonkar S, Raguram A. Ethical dilemmas experienced by clinical psychology trainee therapists. Indian J Med Ethics. 2015;12(4):206-212. (12)*<br>*John TJ, Dharmapalan D. An ethical appraisal of the choice of vaccines against Poliomyelitis. Indian J Med Ethics. 2019;4(1):26-29. (16)*<br>*Basu S, Garg S. Ethical clarity in clinical approaches to rabies prevention in re-exposure cases. Indian J Med Ethics. 2021;VI(1):1-4. (17)*<br>*Das NK, Sil A. Evolution of Ethics in Clinical Research and Ethics Committee. Indian J Dermatol. 2017;62(4):373-379.*	

			(18)<br>*Nimbalkar SM, Patel DS. The Medical Termination of Pregnancy Act: Need to keep pace with technology. Indian J Med Ethics. 2019;4(1):59-64.(21)*<br>*Mukhopadhyay S, Banerjee D. Physician assisted suicide in dementia: A critical review of global evidence and considerations from India. Asian J Psychiatr. 2021;64:102802.(29)*<br>*Singh D, Wadhwa R, Daljit T. Human to Humanoid: An Evolving Concept; Issues and Concerns of Neuroethics. Neurol India. 2022;70(1):25-30. (39)*<br>*Parmar A, Patil V, Sarkar S. Ethical management of substance use disorders: the Indian scenario. Indian J Med Ethics. 2017;2(4):265-270. (41)*<br>*Baru RV, Mohan M. Globalisation and neoliberalism as structural drivers of health inequities. Health Res Policy Syst. 2018;16(Suppl 1):91. Published 2018 Oct 9.(44)*	
**Beneficence**	"Acting in ways that promote the welfare of patients"<br>-clinical research<br><br>*Bibliography*<br>*Różyńska J. Taking the principle of the primacy of the human being seriously. Med Health Care Philos.*	no data	"Prioritizing the welfare of patients"<br>-clinical decision-making<br>-public health (antibiotics, substance use disorders, vaccination, pandemic)<br>-pregnancy risk<br>-clinical psychology	"Creating harm-free environment"<br>-communication with patients<br><br>*Bibliography*<br>*Panaso C. Working experience of nurse anesthetists with*

	2021;24(4):547-562. (113)<br>*Bendowska A, Malak R, Zok A, Baum E. The Ethics of Translational Audiology. Audiol Res. 2022;12(3):273-280. Published 2022 May 13. (22)*<br>*Różyńska J. The ethical anatomy of payment for research participants. Med Health Care Philos. 2022;25(3):449-464. (23)*		-clinical research<br><br>*Bibliography*<br>*Shetty N. Medical Ethics and Law. Indian J Orthop. 2023;57(11):1744-1747. Published 2023 Sep 4. (7)*<br>*Dolma KG, Das M, Saravanabhavan SS, et al. Investigation of an Acute Gastrointestinal Illness Outbreak Linked to Drinking Water in a Higher Educational Institute in East Sikkim, India. Cureus. 2024;16(7):e64050. Published 2024 Jul 7. (8)*<br>*Bhagwat S, Pai SA. Medical ethics in laboratory medicine: A review, with an oath for pathologists. Indian J Med Ethics. 2020;V(1):39-44. (9)*<br>*Bhojaraja MV, Singhai P, Sunil Kumar MM, Sreelatha M. Withdrawal from Dialysis: Why and When? Indian J Palliat Care. 2021;27(Suppl 1):S30-S32. (10)*<br>*Bhola P, Sinha A, Sonkar S, Raguram A. Ethical dilemmas experienced by clinical psychology trainee therapists. Indian J Med Ethics. 2015;12(4):206-212. (12)*<br>*Sarkar MA, Ozair A, Singh KK, Subash NR, Bardhan M, Khulbe Y. SARS-CoV-2 Vaccination in India: Considerations of Hesitancy and Bioethics in Global Health. Ann Glob Health. Ann Glob*	*beneficence for patients. Nurs Ethics. 2024;31(4):508-520. (114)*

			Health. 2021;87(1):124. Published 2021 Dec 10. (14)<br>*Sharma P, Pardeshi G. COVID-19 vaccination in India: An ethical perspective. Diabetes Metab Syndr. 2021;15(6):102314. (15)*<br>*Das NK, Sil A. Evolution of Ethics in Clinical Research and Ethics Committee. Indian J Dermatol. 2017;62(4):373-379. (18)*<br>*Nimbalkar SM, Patel DS. The Medical Termination of Pregnancy Act: Need to keep pace with technology. Indian J Med Ethics. 2019;4(1):59-64.(21)*<br>*Basu S, Garg S. Antibiotic prescribing behavior among physicians: ethical challenges in resource-poor settings. J Med Ethics Hist Med. 2018;11:5. Published 2018 May 12. (34)*<br>*Parmar A, Patil V, Sarkar S. Ethical management of substance use disorders: the Indian scenario. Indian J Med Ethics. 2017;2(4):265-270. (41)*<br>*Rajagopal MR. To comfort always: Are we ignoring this duty in Covid protocols?. Indian J Med Ethics. 2020;V(3):189-191. (83)*<br>*Aneja J, Arora S. Pregnancy and severe mental illness: Confounding ethical doctrines. Indian J*	

			Med Ethics. 2020;V(2):133-139. (101)<br>*Doraiswamy V, Natarajan L, Venkatesh CT. Supraventricular tachycardia in one of the twins: The ethical dilemmas involved in treatment. Ann Pediatr Cardiol. 2020;13(2):150-152. (115)*<br>*Ghoshal R. The social value of research: interrogating the paradoxes. Indian J Med Ethics. 2018;3(1):9-15. (116)*<br>*Sil A, Das NK. Ethics of Safety Reporting of a Clinical Trial. Indian J Dermatol. 2017;62(4):387-391. (117)*	
